# Deciphering the role of CNIH4 in pan-cancer landscapes and its significance in breast cancer progression

**DOI:** 10.3389/fgene.2025.1536620

**Published:** 2025-02-20

**Authors:** Yao Xu, Zengzhen Lai, Chaolin Li

**Affiliations:** Department of Obstetrics and Gynecology, Jinniu Maternity and Child Health Hospital of Chengdu, Chengdu, Sichuan, China

**Keywords:** CNIH4, biomarkers, tumor microenvironment, cell cycle, prognostic value

## Abstract

**Background:**

The escalating global cancer burden necessitates the development of biomarkers with enhanced specificity and sensitivity for early diagnosis and therapeutic efficacy monitoring. The CNIH4 gene, an emerging biomarker, is increasingly recognized for its role in the malignant progression across various cancers.

**Methods:**

We conducted a comprehensive multi-omics analysis of CNIH4, including pan-cancer expression profiles, epigenetic alterations, immune microenvironment characteristics, and therapeutic response patterns. Our focus was on clinical features, molecular underpinnings, and drug sensitivity in breast cancer (BRCA) associated with CNIH4. *In vitro* studies were also performed to assess the effects of CNIH4 knockdown on cell proliferation and cell cycle in the MDA-MB-231 cell line.

**Results:**

CNIH4 upregulation was observed in multiple cancers, significantly correlating with genomic instability. High CNIH4 expression levels were linked to poor prognosis across cancers and associated with key cancer-related pathways, particularly those in cell cycle regulation and DNA repair. Correlation analyses suggest a role for CNIH4 in the tumor immune microenvironment, as evidenced by its association with immune subtypes, immune-related genes, and immune cell infiltration. Single-cell and spatial transcriptome analyses confirmed that CNIH4 expression in BRCA predicts tumor malignancy. Drug sensitivity analysis revealed a significant correlation between CNIH4 and responsiveness to various kinase inhibitors and chemotherapeutic agents. *In vitro* experiments demonstrated that CNIH4 knockdown significantly impacts the proliferation and cell cycle of MDA-MB-231 cells.

**Conclusion:**

Our study highlights CNIH4 as a promising pan-cancer biomarker with significant implications for tumor progression and a critical role in cell cycle regulation in BRCA.

## 1 Introduction

The burgeoning global cancer burden is a critical health concern (Bray et al., 2024). Projections from the American Cancer Society suggest that in 2024, there will be approximately 2 million new cancer cases and 610,000 cancer-related deaths in the United States alone (Siegel et al., 2024). Despite significant advances in therapies such as inhibitory immune checkpoint inhibitors and Chimeric Antigen Receptor T-cell therapy, their efficacy is often hindered by inter-individual variability, tumor heterogeneity, and the complexity of the tumor microenvironment (Suay et al., 2024; Barragan-Carrillo et al., 2025; Ai et al., 2024). Moreover, current diagnostic, prognostic, and therapeutic cancer markers show considerable limitations, with a lack of specificity and sensitivity that impedes the precision of early diagnosis and treatment efficacy monitoring (Zhou et al., 2024). For instance, CA15-3 and CA27-29, key biomarkers for breast cancer (BRCA), suffer from specificity limitations (Seale and Tkaczuk, 2022). Similarly, alpha-fetoprotein (AFP), the most reliable marker for primary liver cancer diagnosis, also faces challenges in sensitivity and specificity (Hu et al., 2022). The intricacies of the tumor microenvironment further complicate the ability of a single marker to accurately reflect the tumor’s biological characteristics and treatment responses. Consequently, there is an urgent need for research to identify novel biomarkers that can enhance the precision and personalization of cancer management. In this context, pan-cancer research has emerged as a vital avenue to address these challenges, offering insights into the intricacies of cancer biology and deepening our understanding of cancer heterogeneity (Li et al., 2023; Chen et al., 2024).

The CNIH4 gene, also known as Cornichon family AMPA receptor auxiliary protein 4, is implicated in various tumorigenesis processes (Fang et al., 2023; Liu and Li, 2022). This gene, part of the Cornichon protein family, is involved in the regulation of G protein-coupled receptor (GPCR) trafficking from the endoplasmic reticulum to the cell surface and in facilitating GPCR export through the early secretory pathway (Sauvageau et al., 2014). The expression of CNIH4 is modulated by TMED9, and research indicates that increased CNIH4 levels are significantly correlated with the malignant progression of multiple cancer types, such as liver hepatocellular carcinoma (LIHC) and colon cancer (Kannangai et al., 2007; Mishra et al., 2019). Within oncology, CNIH4 is emerging as a biomarker that correlates with poor prognosis and enhanced cell proliferation (Zhang et al., 2023). Notably, CNIH4 overexpression is linked to adverse outcomes in low-grade glioma (LGG) and cervical cancer, and its expression in cervical cancer is associated with tumor progression and the inhibition of ferroptosis (Xiao et al., 2023; Wang et al., 2023; Yang et al., 2023). While CNIH4 has been the subject of increasing investigation in specific cancers, comprehensive pan-cancer studies are lacking, which are essential for elucidating the functional relationship between CNIH4 and a broad spectrum of cancers. Understanding this relationship is crucial for advancing our knowledge of tumor heterogeneity and for the development of innovative therapeutic approaches.

This study aims to further explore the relationship between the CNIH4 gene and tumor prognosis and immune microenvironment, to reveal the key role of CNIH4 in tumor development, and to evaluate its potential application in clinical diagnosis and treatment. Through a comprehensive analysis of the function and impact of CNIH4 in various cancers, we aim to provide new strategies for tumor treatment and improve prognosis assessment methods. In particular, this study will focus on the association between CNIH4 and BRCA, aiming to deepen the clinical significance, prognostic value, and functional mechanism of CNIH4 in BRCA, thereby providing new perspectives and strategies for precision medicine and comprehensive management of BRCA.

## 2 Materials and methods

### 2.1 Datasets acquisition

Gene expression profiles of normal human tissues were sourced from the Genotype-Tissue Expression (GTEx) database. Immune cell expression profiles and pan-cancer immunohistochemistry data were procured from the Human Protein Atlas (HPA) database (Jin et al., 2023). Raw and processed RNA-seq data, along with clinical data, were retrieved from the legacy archive of the Genomic Data Commons (GDC) at https://portal.gdc.cancer.gov/legacy-archive/search/f and the Pancancer Atlas publication page (https://gdc.cancer.gov/about-data/publications/pancanatlas). Pan-cancer copy number variation (CNV) and methylation data were accessed via the UCSC XENA website (https://xenabrowser.net/datapages/). The level 4 Simple Nucleotide Variation dataset for all The Cancer Genome Atlas (TCGA) samples was derived from the GDC website (https://portal.gdc.cancer.gov/). Tumor mutation burden (TMB) for each tumor was calculated using the tmb function of the R package maftools (Mayakonda et al., 2018). Pan-cancer Microsatellite instability (MSI) scores, loss of heterozygosity (LOH) scores, homologous recombination deficiency (HRD) scores, and immune subtype data were extracted from prior studies (Bonneville et al., 2017; Thorsson et al., 2018). The BRCA Genome-wide association study (GWAS) dataset was sourced from the Open GWAS website (https://gwas.mrcieu.ac.uk/). The functional states of 14 tumor cells were downloaded from the CancerSEA database (http://biocc.hrbmu.edu.cn/CancerSEA/home.jsp) (Yuan et al., 2019). Protein expression data were obtained from The Cancer Proteome Atlas (TCPA, http://www.tcpaportal.org). Pan-cancer immune cell infiltration data were sourced from the Tumor Immune Estimation Resource 2.0 (TIMER2.0, http://timer.cistrome.org/). Pan-cancer cancer immune cycle data were retrieved from Tracking Tumor Immunophenotype (TIP, http://biocc.hrbmu.edu.cn/TIP/) (Xu et al., 2018). Single-cell transcriptome data were derived from the Tumor Immune Single-cell Hub 2 database (TISCH2, http://tisch.comp-genomics.org/). Spatial transcriptome datasets were obtained from the 10x Genomics server (https://www.10xgenomics.com/) and previous studies (Barkley et al., 2022; Bassiouni et al., 2023). Supplementary Tables S1, S2 provide details of the spatial transcriptome dataset and detailed abbreviations for the 33 tumors.

### 2.2 Evaluation of CNIH4 expression patterns, genomic changes and their prognostic impact in pan-cancer

Gene expression data were derived from the corrected TCGA dataset, with RNA-seq data obtained from the EBPlusPlusAdjustPANCAN_IlluminaHiSeq_RNASeqV2.geneExp.tsv file provided by PanCanAtlas. To expand the sample size, we paired the normal sample TPM expression from GTEx with the TCGA tumor TPM expression (from the tcga_RSEM_gene_tpm and gtex_RSEM_gene_tpm datasets in the UCSC Xena database). To ensure accuracy and account for anatomical factors, only TCGA primary tumor tissues were retained for pairing with GTEx data. The data were converted into unitless Z-score scores by tumor using (x-μ)/σ to standardize the data. Wilcoxon Rank Sum Tests were employed to compare the statistical differences in expression between tumor and normal tissues. The gganatogram package was employed to create organ maps for visualizing the differential expression of CNIH4 between tumor and normal groups in each organ. The pan-cancer expression of CNIH4 was verified using the Gene Expression database of Normal and Tumor tissues 2 (GENT2, http://gent2.appex.kr/gent2/) and the TIMER2.0 database (Park et al., 2019).

A pan-cancer analysis of genomic mutations, amplifications, and deep deletions was conducted using the cBioPortal online server (http://www.cbioportal.org/). Additionally, the CNV and methylation levels of CNIH4, along with their correlation with mRNA expression, were assessed based on pan-cancer CNV and methylation data. To identify clinically relevant alternative splicing (AS) events, the ClinicalAS module of the OncoSplicing website (http://www.oncosplicing.com/) was used (Zhang et al., 2022). All AS events from the SplAdder and SpliceSeq projects were included, and the percent spliced-in (PSI) differences of AS events between normal and tumor tissues, as well as their associations with clinical outcomes, were compared. Co-localization analysis of Expression Quantitative Trait Loci-Genome-Wide Association Study (eQTL-GWAS) was performed using a Bayesian co-localization approach. The co-localization evidence cutoff was set at PP.H4.abf >80%, and results were visualized using stack_assoc_plot from the gassocplot2 package (Zhou et al., 2023).

Receiver operating characteristic (ROC) analysis was conducted using the pROC package, calculating the 95% confidence interval, total area under the curve, and smooth ROC curve to evaluate the diagnostic performance of CNIH4 gene expression for tumor and normal groups. Univariate Cox survival analysis and Kaplan-Meier (KM) survival analysis were performed using the survival package in R to assess CNIH4 expression as a predictor of overall survival (OS), disease-specific survival (DSS), progression-free interval (PFI), and disease-free interval (DFI). Optimal cutoff values for high-expression and low-expression groups were determined using the survminer package, and the log-rank test was performed using the survfit function to evaluate the significance between groups. The restricted cubic spline (RCS) method was employed to explore non-linear effects of CNIH4 on survival risk. Patients were stratified into four groups (Q1, Q2, Q3, Q4) based on CNIH4 expression, with Q1 representing the top 25% of samples with the highest expression and Q4 the bottom 25% with the lowest. The chi-square test was used to detect significant differences in patient composition across groups. Univariate and multivariate Cox proportional hazard regression analyses were conducted to identify potential independent prognostic factors, and the “forestplot” package was used to create forest plots. Based on the multivariate Cox proportional hazard model results, the “rms” package was utilized to construct a nomogram predicting 1-year, 2-year, and 3-year OS rates for clinical patients (Li et al., 2023).

### 2.3 Gene set enrichment analysis (GSEA)

Based on the functional states of 14 tumor cell types cataloged by the CancerSEA database, we employed the z-score parameter in the R package GSVA to calculate the z-scores for these 14 functional state gene sets, thereby deriving a composite z-score for each (Hänzelmann et al., 2013; Lee et al., 2008). The scale function was subsequently applied to standardize these scores into gene set scores. We then computed the Pearson correlation coefficients between the CNIH4 gene and each gene set score. Tumor samples were stratified into high and low CNIH4 expression groups based on the median expression value of CNIH4. The limma package was utilized for differential expression analysis, yielding log2 fold change (log2FC) values for each gene. Thereafter, the GSEA function within the clusterProfiler package was employed to conduct gene set enrichment analysis with both the hallmark gene set and the KEGG metabolic gene set (Yu et al., 2012). This analysis generated normalized enrichment scores (NES) for each gene set, performed significance testing and multiple hypothesis testing on the NES values, and visualized the results using a bubble chart. Protein expression data were sourced from the TCPA database. Pathway activity scores for 10 cancer-related pathways—including TSC/mTOR, RTK, RAS-MAPK, PI3K-AKT, hormone ER, hormone AR, EMT, DNA damage response, cell cycle, and apoptosis—were ascertained based on published studies (Liu et al., 2023). The wilcox.test function was applied to assess the differences in pathway activity scores between the high and low CNIH4 expression groups. Additionally, we conducted a differential expression analysis to further elucidate the potential pathways and molecular mechanisms associated with CNIH4 in BRCA. Tumor samples were collected from breast cancer patients and categorized into high and low-expression groups based on the median expression level of CNIH4. Differential analysis was executed utilizing the limma package, with genes exhibiting |logFC| > 0.585 and a corrected p-value of less than 0.05 classified as significantly different. Volcano plots were generated for visualization. Furthermore, functional enrichment analysis was performed using the Metascape online platform (https://metascape.org/gp/index.html#/main/step1).

### 2.4 Immune correlation analysis

Utilizing pan-cancer immune expression profile data, we assessed the distribution of various immune subtypes between high and low-expression groups of CNIH4. Pearson correlation analysis was conducted on each immune gene with CNIH4 using the cor.test function, and the resulting data were visualized as a heatmap with the ComplexHeatmap package in R. The R package ESTIMATE was employed to determine the stromal and immune scores, as well as the ESTIMATE scores, for each patient’s tumor based on CNIH4 gene expression levels (Yoshihara et al., 2013). The XCELL algorithm assessed the correlation between CNIH4 gene expression and immune cell infiltration (Aran et al., 2017). Furthermore, the TIP database provided quantification of the scores for the seven steps of the cancer immune cycle across various cancers. Spearman correlation analysis was applied to calculate the correlations between individual genes and TIP scores.

### 2.5 Single-cell and spatial transcriptome analysis

Pan-cancer single-cell resolution expression data for genes in BRCA were sourced from the TISCH2 database. We employed the pheatmap package to construct heatmaps visualizing the pan-cancer single-cell expression landscape of these genes. Uniform Manifold Approximation and Projection (UMAP) technology was utilized to delineate distinct cell populations, with a focus on visualizing and analyzing the expression data of the CNIH4 gene. Cells were categorized into CNIH4 expression-positive and -negative groups, and the proportion of each cell type within these groups was calculated. The AUCell package was applied to assess the scores of various biological pathways, including immune, metabolic, signaling, proliferation, cell death, and mitochondrial-related processes. The limma package was used to compare the pathway scores between the CNIH4 expression-positive and -negative groups.

Drawing on previous studies, the Cottrazm package was used for deconvolution analysis of cell composition in spatial transcriptome sections, identifying the predominant cell type in each microregion (Shi et al., 2024; Xun et al., 2023). The SpatialPlot function from the Seurat package was employed for visualization purposes. Data were normalized using the scale function for Z-score standardization, with pheatmap used for subsequent visualization. We examined the average expression of the CNIH4 gene across cell types in each section. The SpatialFeaturePlot function in the Seurat package visualized the expression landscape of the CNIH4 gene within each microregion. Spearman correlation analysis was conducted to determine the correlations between cell content across all spots, as well as between cell content and gene expression. The linkET package was utilized for visualizing these correlations.

### 2.6 Drug sensitivity analysis

We employed Spearman correlation analysis to determine the correlation between gene expression and dose-response curve (area under the curve - AUC) values within the Cancer Therapeutics Response Portal (CTRP) and Profiling Relative Inhibition Simultaneously in Mixtures (PRISM) databases (Rees et al., 2016; Corsello et al., 2020). A negative correlation indicates increased drug sensitivity with higher gene expression levels, while a positive correlation suggests greater drug resistance. Additionally, we assessed the correlation between CNIH4 expression and the half-inhibitory concentration (IC50) of various drugs in cancer cell lines, utilizing expression data and drug information from the CellMiner database (Reinhold et al., 2012).

### 2.7 Cell culture

The human BRCA cell lines HBL-100 and MDA-MB-231 were procured from Pricella Biotechnology in Wuhan, China. MDA-MB-231 cells were cultured in Dulbecco’s Modified Eagle Medium (DMEM, HyClone, USA) supplemented with 10% fetal bovine serum (FBS, Vazyme, China), 100 mg/mL streptomycin, and 100 U/mL penicillin. HBL-100 cells were maintained in Roswell Park Memorial Institute 1,640 Medium (RPMI-1640, Gibico, USA) with the same supplements. Both cell lines were incubated in a humidified atmosphere containing 5% CO2 at a temperature of 37°C.

### 2.8 Quantitative real-time PCR (qRT-PCR)

Total RNA was extracted using TRIzol reagent (Invitrogen, USA) according to the manufacturer’s protocol. One microgram of RNA was reverse transcribed into cDNA using the HiScript III 1st Strand cDNA Synthesis Kit (Vazyme, China). Glyceraldehyde 3-phosphate dehydrogenase (GAPDH) was amplified from each sample to verify equal cDNA input. Each PCR reaction comprised 1 μL of cDNA, 0.6 μL of each forward and reverse primer (10 μM), 7.5 μL of ChamQ Universal SYBR qPCR Master Mix (Vazyme, China), and 6.3 μL of ddH2O. The PCR cycling conditions were as follows: initial denaturation at 95°C for 10 min, followed by 40 cycles of denaturation at 95°C for 15 s, annealing at 62°C for 1 min, and extension at 72°C for 15 s. A final extension step was conducted at 60°C for 1 min and 95°C for 15 s. The primer sequences for GAPDH were forward: 5′-GGA​GCG​AGA​TCC​CTC​CAA​AAT-3′ and reverse: 5′-GGC​TGT​TGT​CAT​ACT​TCT​CAT​GG-3'. The primer sequences for CNIH4 were forward: 5′-TCA​ACT​TAC​CTG​TTG​CCA​CTT​G-3′ and reverse: 5′-TCT​GTT​GGA​TCA​AAC​ACT​CCC​A-3'.

### 2.9 Western blotting

Following siRNA treatment, cells were harvested and collected via centrifugation after triple washing with phosphate-buffered saline (PBS). Total protein extracts were prepared by supplementing RIPA buffer with protease inhibitors (Solarbio, China). Western blot analysis was conducted using primary antibodies against CNIH4 (Santa Cruz, USA) and GAPDH (Proteintech, China), according to the manufacturer’s protocols. Goat Anti-Mouse IgG-HRP (Proteintech, China) and Goat Anti-Rabbit IgG-HRP (Proteintech, China) served as secondary antibodies. GAPDH was employed as a protein loading control. Signal detection was achieved using an enhanced chemiluminescence (ECL) reagent (4A Biotech, China).

### 2.10 Flow cytometric analysis of cell cycle

The cell cycle distribution of MDA-MB-231 cells was assessed using the Cell Cycle Detection Kit (KeyGen Biotech, China). Briefly, cells were collected and fixed in 70% cold ethanol at 4°C overnight. Following two washes with PBS, the cells were incubated with a PI/RNase staining buffer for 30 min. The stained cells were then analyzed using a Beckman flow cytometer and CytExpert Software.

### 2.11 EdU cell proliferation assay

The proliferation of MDA-MB-231 cells was assessed using the EdU (5-ethynyl-2′-deoxyuridine) cell proliferation assay, following the manufacturer’s protocol. Approximately 1 × 10^5 cells were seeded in 12-well plates and cultured for 24 h before the assay. A total of 500 μL of EdU reagent (10 μM, Beyotime, C0071S) was added to each well and incubated for 2 h to label the proliferating cells. After three washes with PBS, cells were fixed in a 4% paraformaldehyde solution (Dingguo Biotechnology, AR-0211) for 15 min, permeabilized with 0.3% Triton X-100 (GenStar, VA11410) for an additional 15 min, and then incubated with the click-reaction reagent for 30 min at room temperature in the dark. A 1× Hoechst 33,342 reagent was used to counterstain the nucleus. Staining results were observed using a Nikon ECLIPSE Ti-S fluorescence microscope system, and data were collected with NIS-Elements F v4.0 software.

### 2.12 Statistical analysis

Statistical analyses were conducted using R version 4.2.1 in conjunction with relevant online databases. The unpaired Wilcoxon Rank Sum Test and Signed Rank Test were applied to assess the significance of differences between paired groups, while the Kruskal–Wallis test (kruskal.test) was utilized to evaluate differences among multiple sample groups. Survival analyses were performed using KM curves, with log-rank tests or Cox proportional hazards regression models serving as supplementary statistical measures. Pearson or Spearman correlation coefficients were calculated to determine the strength and direction of relationships between variables. All cellular experiments were conducted in triplicate. Data from cellular experiments were analyzed using GraphPad Prism for Windows (version 9.0.0). Statistical significance was set at *p* < 0.05, with *p* < 0.05 indicated by one asterisk (*), *p* < 0.01 by two asterisks (**), *p* < 0.001 by three asterisks (***), and ‘ns' denoting not significant.

## 3 Results

### 3.1 CNIH4 is significantly upregulated in multiple cancers

**FIGURE 1:**
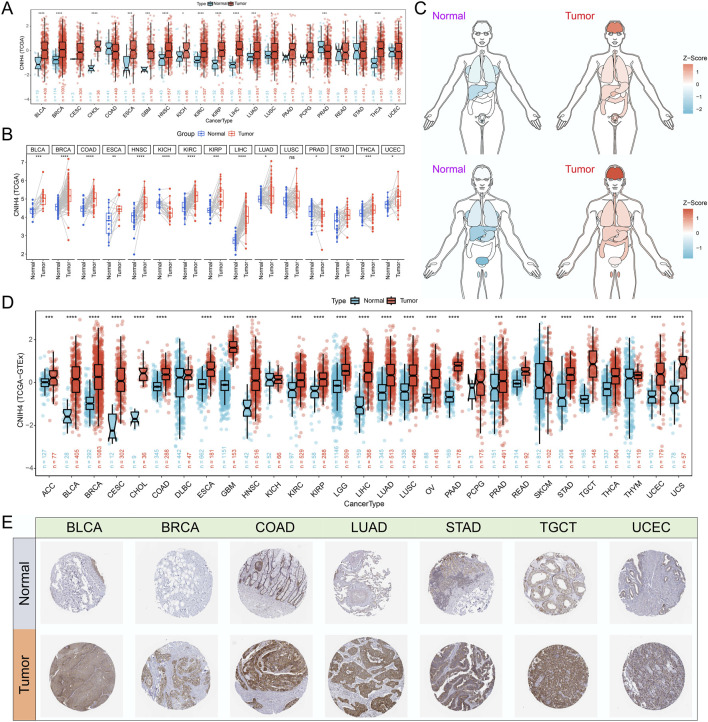
**(A)** Evaluation of the differential expression of CNIH4 in pan-cancer using TCGA tumor and normal samples. The upper and lower ends of the box represent the interquartile range of the values. The line in the box represents the median. Wilcoxon Rank Sum Tests were used to compare the statistical differences in expression between the two groups; **(B)** Assessment of the pan-cancer differential expression of CNIH4 based on paired TCGA tumor and normal samples. Paired samples were connected by lines. Wilcoxon signed-rank test was used to compare expression levels between two groups; **(C)** Pan-cancer organ map illustrating the differential expression of CNIH4 derived from TCGA and GTEx. Blue means Z score is less than 0, red means Z score is greater than 0, and the darker the color, the larger the absolute value of Z score; **(D)** Box plot demonstrating the pan-cancer differential expression of CNIH4 based on TCGA and GTEx The upper and lower ends of the box represent the interquartile range of the values. The line in the box represents the median. Wilcoxon Rank Sum Tests were used to compare the statistical differences in expression between the two groups; **(E)** Analysis of the differential protein expression of CNIH4 in pan-cancer utilizing the HPA database. *p < 0.05, **p < 0.01, ***p < 0.001, ****p < 0.0001, ns: not significant.

Analysis based on the GTEx database revealed that CNIH4 expression was elevated in normal human tissues, including skeletal muscle, esophagus, and adipose tissue (Supplementary Figure 1A). Examination of immune cell expression profiles indicated that CNIH4 was markedly overexpressed in various monocyte subtypes (Supplementary Figure 1B). Differential expression analysis using TCGA data showed that CNIH4 was significantly upregulated in bladder urothelial carcinoma (BLCA), BRCA, and esophageal carcinoma (ESCA) cancers (Figures 1A, B). To augment the sample size, we integrated data from the GTEx database. An organ map depicted that CNIH4 was dysregulated and generally overexpressed in tumors of the liver, stomach, breast, intestine, and brain (Figure 1C). Furthermore, box plots revealed significantly high CNIH4 expression across 26 cancer types, including BRCA (Figure 1D). Verification using the GENT2 and TIMER2.0 databases confirmed the significant overexpression of CNIH4 in multiple tumors (Supplementary Figures 1C, D). Additionally, immunohistochemical analysis from the HPA database showed that CNIH4 protein was markedly overexpressed in various tumor types, including BLCA, BRCA, and lung adenocarcinoma (LUAD) (Figure 1E; Supplementary Figure 1E).

### 3.2 Genomic variations, epigenetic alterations, and AS events of CNIH4 in pan-cancer

**FIGURE 2:**
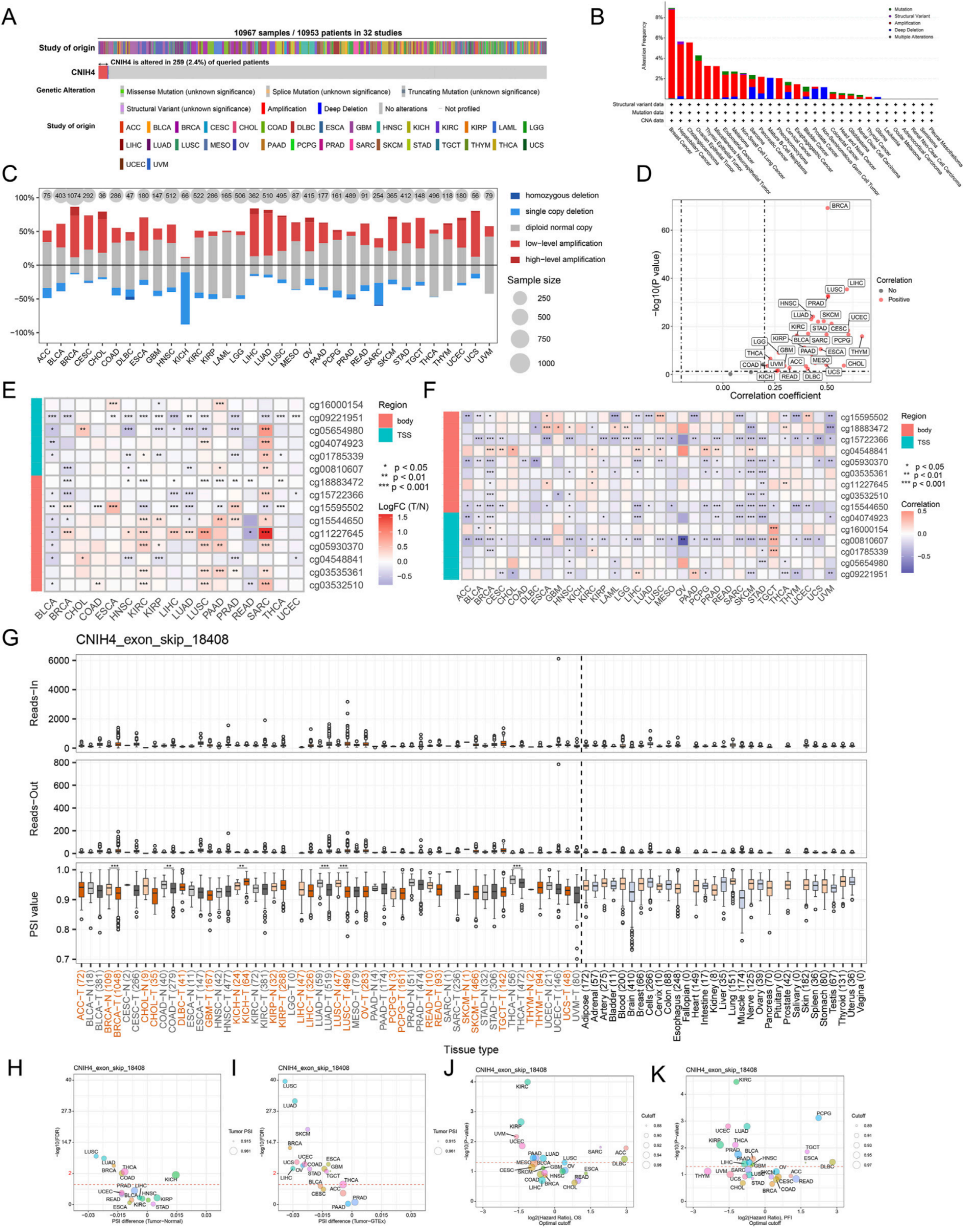
**(A)** Assessment of the pan-cancer mutational landscape of CNIH4 using the cBioPortal database; **(B)** Mutation frequency of CNIH4 across various cancer types. The mutation frequency is represented by the height of the bar graph. Different bar colors indicate different mutation types (green: Mutation; purple: Structural Variant; red: Amplification; blue: Deep Deletion; gray: Multiple Alterations).; **(C)** Levels of copy number variation for CNIH4 in pan-cancer; **(D)** Correlation between copy number variation and mRNA levels of CNIH4 in pan-cancer; **(E)** Differential methylation levels of CNIH4 in pan-cancer; **(F)** Correlation between methylation levels and mRNA expression of CNIH4 in pan-cancer; **(G)** Alternative splicing events involving CNIH4 in pan-cancer. *p < 0.05, **p < 0.01, ***p < 0.001; **(H–K)** percent spliced-in (PSI) differences when comparing tumors with corresponding healthy or adjacent tissues and the association between CNIH4 exon_skip_18408 events and prognosis.

Analysis utilizing the cBioPortal server revealed that CNIH4 mutations were present in 259 of 10,953 patients, with the highest mutation rate observed in BRCA, predominantly consisting of amplification mutations (Figures 2A, B). CNV analysis indicated a prevalence of amplification mutations in CNIH4 across multiple cancers, including BRCA, colon adenocarcinoma (COAD), and LGG. In contrast, deletion mutations were more frequent in kidney chromophobe (KICH) and sarcoma (SARC) (Figure 2C). Further analysis of the TCGA-BRCA dataset highlighted CNV across multiple chromosomes, with specific regions such as ‘chr1′, ‘chr8′, and ‘chr17′exhibiting higher GISTIC scores, suggesting a potential role in BRCA (Supplementary Figure 2A). CNIH4 expression in BRCA showed an upward trend with increasing copy number, ranging from homozygous deletion to high copy number amplification (Supplementary Figure 2B). Samples with the highest 25% CNIH4 expression in BRCA exhibited a higher fraction of genomic alterations, indicating increased proliferation activity, genomic instability, and heterogeneity (Supplementary Figure 3C). Correlation analysis demonstrated a significant positive correlation between CNIH4 mRNA expression and CNV in multiple cancers, including BRCA (Figure 2D; Supplementary Figure 2D). Epigenetic alteration analysis revealed dysregulation of CNIH4 methylation in multiple tumors compared to normal tissues (Figure 2E). A significant negative correlation was observed between CNIH4 mRNA expression and methylation levels in various methylation probes in pan-cancer (Figure 2F; Supplementary Figures 3A–C). Survival analysis associated CNIH4 hypomethylation with poor prognosis in cancers such as adrenocortical carcinoma (ACC), LIHC, and glioblastoma multiforme (GBM) (Supplementary Figure 3D).

Using OncoSplicing, we identified 36 clinically relevant AS events of the CNIH4 gene, detailed in Supplementary Tables S3. These include the CNIH4_AA-9954 event from the SpliceAdderSeq project and the CNIH4_exon_skip_18408 event from the SpliceSeq project. Figure 2G illustrates the pan-cancer analysis of the CNIH4_exon_skip_18408 event’s PSI values. Notably, reduced PSI scores were observed in tumor tissues from BRCA, COAD, LUAD, lung squamous cell carcinoma (LUSC), and thyroid carcinoma (THCA) compared to normal samples, while elevated PSI scores were noted in tumor tissues from KICH (Figures 2G–I). Furthermore, significant associations between PSI scores of CNIH4 and clinical prognosis across various cancers were identified (Figures 2J, K). Detailed information regarding the CNIH4_AA-9954 event can be found in Supplementary Figures S4A–D. These findings suggest that AS events of CNIH4 significantly influence the progression of numerous cancers. Moreover, we discovered that the mRNA expression of CNIH4 in pan-cancer was significantly correlated with TMB, MSI, LOH, and HRD scores (Supplementary Figures 4E–H). Collectively, these data underscore an important association between CNIH4 and genomic instability.

### 3.3 Evaluation of the diagnostic and prognostic value of CNIH4 in pan-cancer

**FIGURE 3:**
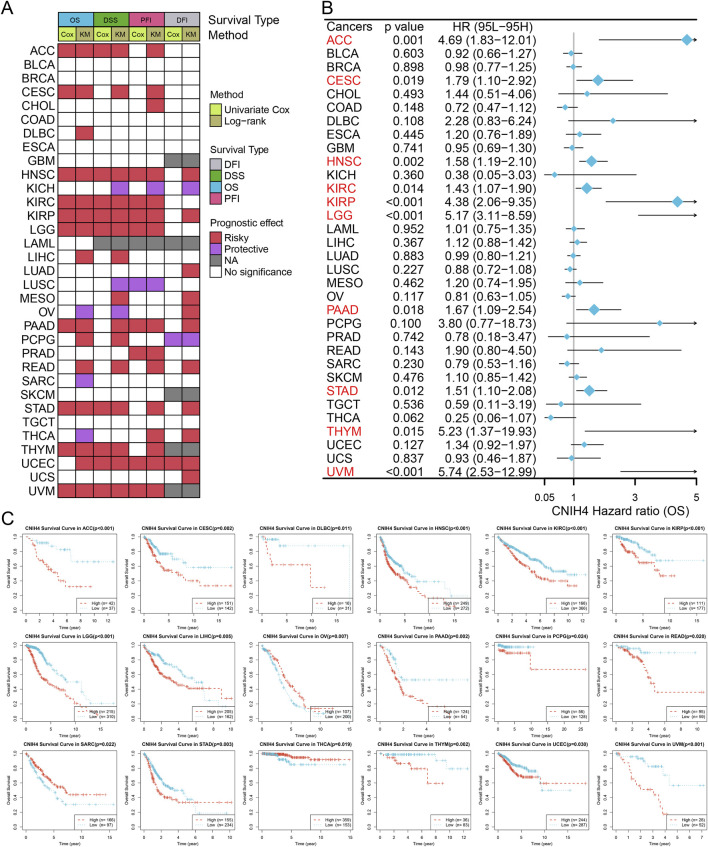
**(A)** Summarize the results of two tests for four types of survival in the TCGA database. For each survival period, the Unicox and km methods were used to calculate respectively. Red represents at risk, green represents protection, grey represents no calculation, and white represents no significance; **(B)** Univariate Cox survival analysis between CNIH4 and overall survival in Pan-Cancer. A risk ratio greater than 1 indicates an increased risk, while a risk ratio less than 1 indicates a decreased risk. Error bars represent 95% confidence intervals; **(C)** Kaplan-Meier survival analysis between CNIH4 and overall survival in Pan-Cancer. Red represents the high expression group, blue represents the low expression group, and the Log-rank test examines the difference in the survival curves of the two groups. P less than 0.05 is considered significant.

The ROC curve showed that CNIH4 has the potential to become a diagnostic biomarker for multiple tumors including BRCA (Supplementary Figure 5). Figure 3A summarizes the results of survival analysis based on Univariate Cox regression analysis and KM curve. The results of univariate Cox survival analysis showed that CNIH4 was a risk factor for multiple tumors including head and neck squamous cell carcinoma (HNSC), kidney renal clear cell carcinoma (KIRC), kidney renal papillary cell carcinoma (KIRP), LGG, and uveal Melanoma (UVM), that is, patients with tumors in the CNIH4 high expression group had a shorter OS, DSS, and PFI. In addition, there were different degrees of correlation with multiple tumors including cervical squamous cell carcinoma and endocervical adenocarcinoma (CESC), stomach adenocarcinoma (STAD), thymoma (THYM), uterine corpus endometrial carcinoma (UCEC), and LGG. For DFI, we observed that CNIH4 was a risk factor for UCEC, but a protective factor for pheochromocytoma and paraganglioma (PCPG). In addition, the KM survival curve also showed that high expression of CNIH4 was significantly associated with poor prognosis in multiple tumors (Figures 3B, C; Supplementary Figures 6A–S; Supplementary Figures 7A–N; Supplementary Figures 8A–R).

Notably, significant associations between CNIH4 and various survival durations in UVM were observed and further analyzed. RCS analysis confirmed a linear effect of CNIH4 on UVM OS (Supplementary Figure 9A). A chi-square test also validated that the highest number of deaths occurred among the 25% of UVM patients with the highest CNIH4 expression (Supplementary Figure 9B). Time-dependent ROC analysis revealed that the AUCs for CNIH4 in predicting 1-year, 2-year, and 3-year survival in UVM patients were 0.82, 0.79, and 0.80, respectively (Supplementary Figure 9C). A risk factor heatmap further suggested a higher number of deaths in patients with elevated CNIH4 expression (Supplementary Figure 9D). This finding was corroborated in additional datasets (Supplementary Figure 9E). Univariate and multivariate Cox analyses established CNIH4 as an independent prognostic factor for UVM (Supplementary Figures 9F, G). Based on the multivariate Cox analysis, a nomogram was constructed to predict the 1-year, 2-year, and 3-year survival rates of UVM patients. Calibration curve analysis demonstrated the prediction model’s high accuracy in forecasting the survival rates of UVM patients at 1-year, 2-year, and 3-year intervals (Supplementary Figures 9H, I).

### 3.4 CNIH4 is significantly associated with multiple cancer-related pathways

**FIGURE 4:**
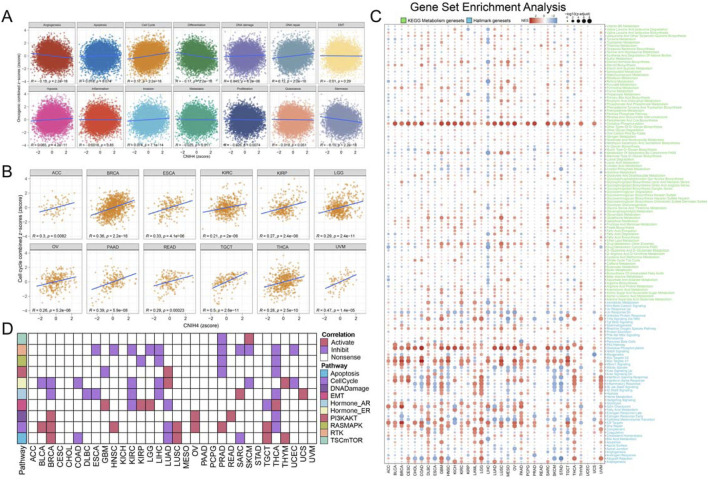
**(A)** Pearson correlation analysis between pan-cancer malignant feature score and CNIH4. The ordinate is the zscore standardized score of the combined z-score value of each functional state, the abscissa is the zscore of gene expression, different colors represent different functional state types, and R is the coefficient of Pearson correlation analysis; **(B)** Pearson correlation analysis between pan-cancer malignant cell cycle score and CNIH4; **(C)** Gene set enrichment analysis was performed on the Hallmark gene set and the KEGG metabolic gene set in Pan-Cancer using the GSEA function in the clusterProfiler package. Negative NES indicates that the target pathway is significantly enriched in the low expression group, and *vice versa*. Darker colors represent higher absolute values of enrichment scores. The bubble size reflects the adjusted p-value.; **(D)** Differences in TCPA pathway activity. In the analysis, red indicates that pathway activity is elevated in the high expression group, green signifies that pathway activity is diminished in the high expression group, and white denotes no significant difference.

We analyzed to evaluate the correlation between CNIH4 and 14 tumor-related pathways obtained from the CancerSEA database. The findings indicated that in pan-cancer, CNIH4 exhibited a positive correlation with cell cycle and DNA repair pathways to a moderate extent, and a negative correlation with angiogenesis and differentiation pathways to a similar extent (Figure 4A). Further analysis of the relationship between CNIH4 and cell cycle pathways revealed significant positive correlations in multiple cancers, including ACC, BRCA, and ESCA (Figure 4B). GSEA demonstrated that CNIH4 was significantly and positively correlated with cell cycle pathways such as Myc Targets V1 and E2F Targets across various cancers. Additionally, CNIH4 showed varying degrees of correlation with pathways like Oxidative Phosphorylation, Epithelial Mesenchymal Transition, and Inflammatory Response in multiple tumors (Figure 4C). Proteomic functional enrichment analysis was also performed, revealing that the high CNIH4 expression group displayed increased activity in the cell cycle pathway in several cancers, including BRCA. Furthermore, the high CNIH4 expression group in LIHC, pancreatic adenocarcinoma (PAAD), and THYM exhibited decreased activity in the RAS-MAPK and Receptor Tyrosine Kinase (RTK) pathways (Figure 4D).

### 3.5 Immune signature of CNIH4 in pan-cancer

**FIGURE 5:**
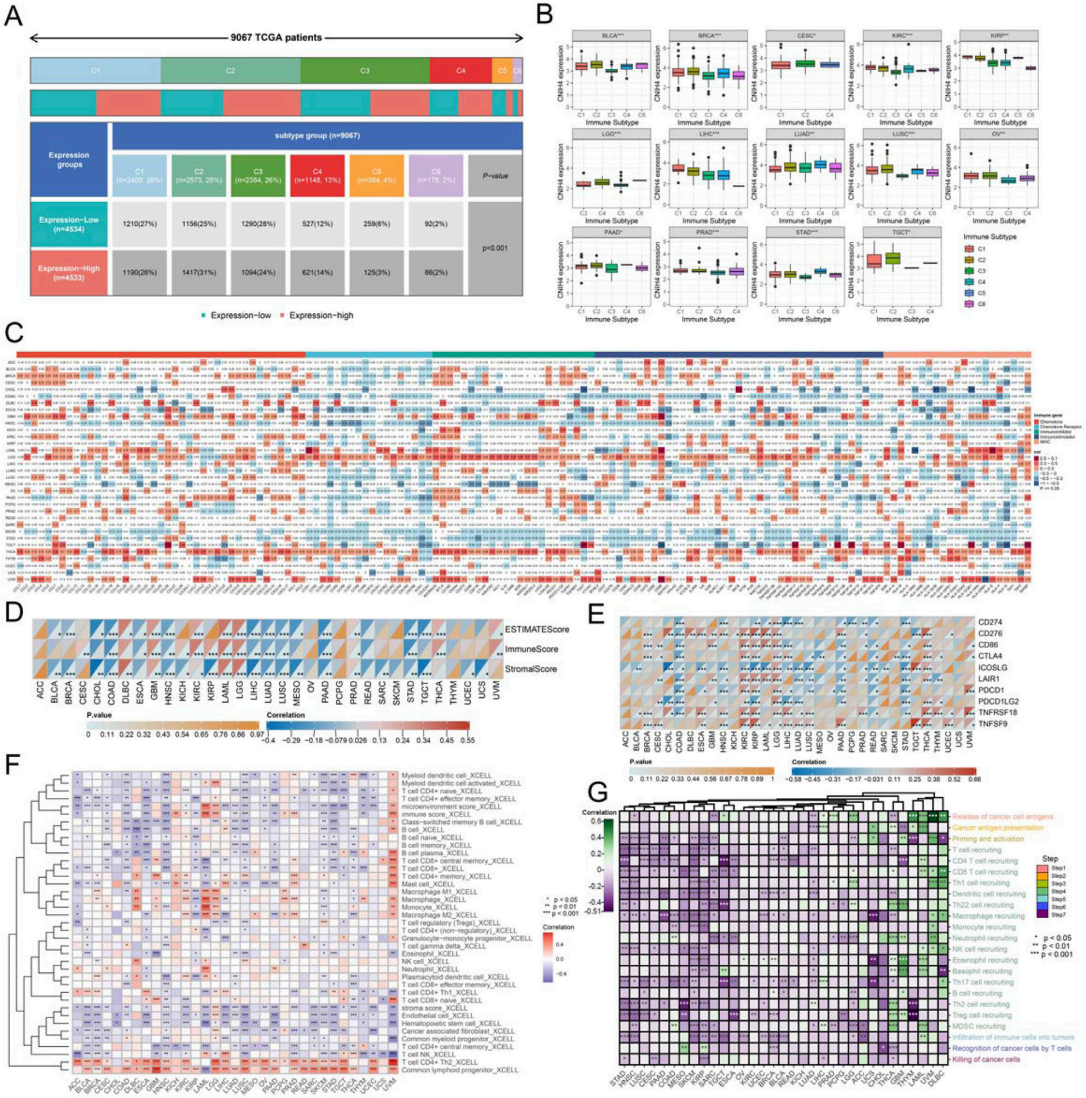
**(A, B)** Differential expression of CNIH4 among different immune subtypes in pan-cancer. The top bar graph shows the proportion of each subtype across all samples. The second row illustrates the proportions of high-expression (red) and low-expression (green) gene groups for each subtype. The bottom section displays the number and proportion of each subtype within each group; **(C)** Pearson correlation analysis between pan-cancer immune genes and CNIH4. The intensity of red indicates a stronger positive correlation, while a deeper blue signifies a stronger negative correlation; **(D)** ESTIMATE algorithm evaluates the correlation between CNIH4 and immune and stromal scores in pan-cancer; **(E)** Correlation analysis between pan-cancer immune checkpoint genes and CNIH4; **(F)** XCELL algorithm evaluates the correlation between CNIH4 and immune cell infiltration in pan-cancer; **(G)** Spearman correlation between TIP score and CNIH4 gene expression. *p < 0.05, **p < 0.01, ***p < 0.001.

Pan-cancer immune subtype analysis revealed that the C2 subtype, characterized by IFN-γ dominance, was predominant in the group with high CNIH4 expression, while the C3 subtype, associated with inflammation, was the main immune subtype in the low expression group (Figures 5A, B). Correlation analysis indicated that CNIH4 was significantly positively correlated with immune-related genes in LGG, THCA, and UVM, and significantly negatively correlated with immune-related genes in COAD, skin cutaneous melanoma (SKCM), and HNSC (Figure 5C). Furthermore, CNIH4 showed a significant negative correlation with ImmuneScore and StromalScore in multiple cancers, including COAD and LIHC, while it was significantly positively correlated with LGG, KIRC, and acute myeloid leukemia (LAML) (Figure 5D). CNIH4 was also observed to be significantly positively correlated with multiple immune checkpoints, including CD276, CD86, and PDCD1, in cancers such as KIRC, KIRP, and LGG (Figure 5E). Immune cell infiltration analysis using the XCELL algorithm demonstrated that CNIH4 was significantly positively correlated with T cell CD4^+^ Th2 and common lymphoid progenitor cell infiltration and negatively correlated with microenvironment score, endothelial cells, hematopoietic stem cells, T cell CD4^+^ central memory, T cell CD4^+^ naïve, T cell CD8^+^, and cancer-associated fibroblasts to varying degrees (Figure 5F; Supplementary Figures 10A–I). The anti-cancer immune status, as reflected in the cancer-immune cycle, showed that CNIH4 was significantly negatively correlated with priming and activation (Step 3), trafficking of immune cells to tumors (Step 4), and infiltration of immune cells into tumors (Step 5) in most cancers, and significantly positively correlated with the release of cancer cell antigens (Step 1) in cancers including LAML, diffuse large B-cell lymphoma (DLBC), and LGG (Figure 5G).

### 3.6 CNIH4 and BRCA are significantly associated with malignant cells

**FIGURE 6:**
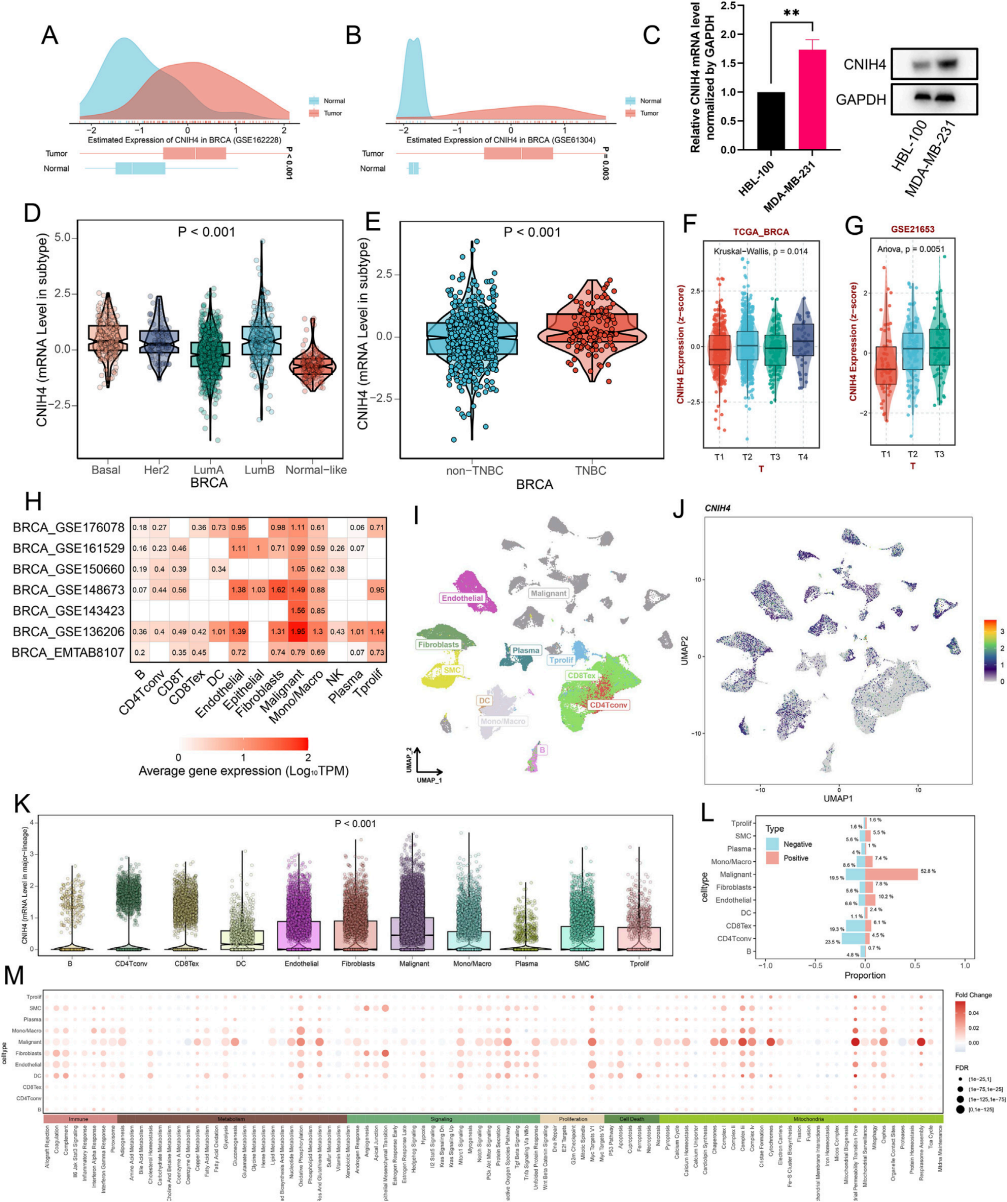
**(A, B)** Validation of high expression of CNIH4 in BRCA based on data from GEO dataset. The distribution of gene expression levels in tumor and normal groups is shown above the figure. The box plot’s edges represent the interquartile range, and the line inside the box indicates the median; **(C)** Compared with normal cells HBL-100, CNIH4 is significantly overexpressed in the BRCA cell line MDA-MB-231; **(D)** Expression of CNIH4 in BRCA subtypes. The upper and lower boundaries of the box represent the interquartile range of the values, while the line within the box indicates the median; **(E)** CNIH4 is significantly overexpressed in TNBC; **(F, G)** CNIH4 is more highly expressed in BRCA high clinical stage; **(H)** CNIH4 expression is higher in malignant cells at the single cell level; **(I)** UMAP of main cell lineage in BRCA_GSE176078; **(J)** UMAP of CNIH4 in BRCA_GSE176078. The color scale on the right side of the graph represents the expression level of the gene. Each dot in the graph represents a cell; **(K)** Differential expression of CNIH4 gene among cells in BRCA_GSE176078. The horizontal axis represents various cell types, each identified by a distinct color. The vertical axis denotes the gene expression values. Each scattered point corresponds to an individual cell; **(L)** The proportion of each cell type in the CNIH4 gene expression positive group and negative group in BRCA_GSE176078. Red and blue indicate the positive and negative groups of CNIH4 gene expression, respectively. The y-axis represents cell types, while the x-axis shows the proportion of each cell within the group, with bar length corresponding to this proportion; **(M)** Differences in pathways of various cell types in the positive/negative groups of CNIH4 gene expression in BRCA_GSE176078. The y-axis denotes various cell types, and the x-axis indicates different pathways. Red signifies an increase in pathway score (activation) in the CNIH4 expression positive group, while blue denotes a decrease (inhibition). Bubble size reflects result significance.

Analysis of the GEO database dataset confirmed the significantly elevated expression of CNIH4 in BRCA (Figures 6A, B). Comparative expression analysis of CNIH4 in normal and BRCA cells revealed that, in contrast to the normal cell line HBL-100, the MDA-MB-231 cell line exhibited significantly higher CNIH4 expression at both the mRNA and protein levels (Figure 6C). Subtype analysis indicated that CNIH4 was predominantly and significantly overexpressed in the LumB subtype of BRCA (Figure 6D). Additionally, we observed significantly higher CNIH4 expression in triple-negative breast cancer (TNBC), which is associated with a higher degree of malignancy (Figure 6E). Elevated CNIH4 expression was also noted in advanced-stage BRCA (Figures 6F, G). Co-localization analysis of eQTL-GWAS data identified a common genetic causal variant at the single nucleotide polymorphism (SNP) site rs4654036 shared between BRCA and CNIH4 gene expression (Supplementary Figures 11A–C).

**FIGURE 7:**
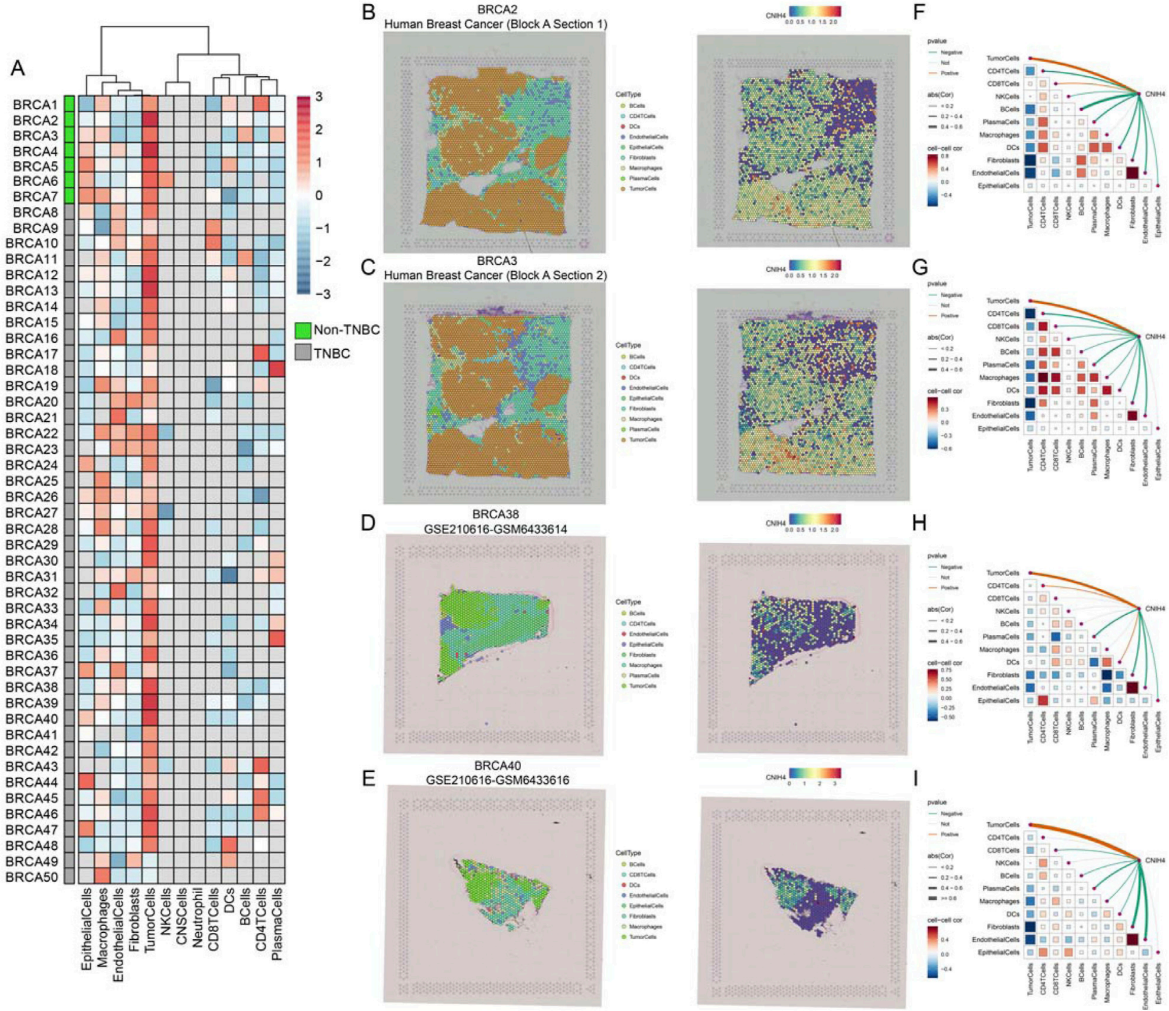
**(A)** Expression of CNIH4 gene in each microdomain in BRCA spatial transcriptome sections; **(B–E)** The cell type with the largest proportion in each microdomain at BRCA idle resolution and the spatial transcriptome localization of the CNIH4 gene. Each dot is a spot for spatial transcriptome sequencing, and different colors represent different cell types. The darker the color (red) in the same spot, the higher the expression of the CNIH4 gene in the spot; **(F–I)** Spearman correlation of CNIH4 gene expression with each cell type in microdomains at idle resolution. The red line indicates a positive correlation, the green line denotes a negative correlation, the gray line signifies no statistical significance, and the thickness of the line reflects the absolute value of the correlation coefficient.

Single-cell analysis demonstrated that CNIH4 was predominantly highly expressed in malignant cells of BRCA, followed by monocytes and macrophages (Figure 6H). UMAP mapping and expression analysis of the BRCA_GSE176078 dataset further validated the significantly elevated CNIH4 expression in malignant cells (Figures 6I–K). Cell ratio analysis of the BRCA_GSE176078 dataset revealed that the proportion of malignant cells in the CNIH4-positive expression group was substantially higher than that in the CNIH4-negative expression group (Figure 6L). Pathway analysis in malignant cells showed that the Metabolism, Proliferation, and Mitochondria-related biological pathways were more active in the CNIH4-positive group (Figure 6M). Spatial transcriptome resolution analysis in multiple BRCA spatial transcriptome sections indicated that CNIH4 expression was more pronounced in malignant cell microregions (Figure 7A). Single gene localization analysis revealed that CNIH4 expression was similarly localized to tumor cells, suggesting that in BRCA, CNIH4 may be primarily expressed by tumor cells (Figures 7B–E). Correlation analysis was highly consistent with the localization results, showing that CNIH4 expression levels were significantly and positively correlated with the tumor cell content in the spot (Figures 7F–I).

### 3.7 Knockdown of CNIH4 in BRCA significantly inhibits cell cycle and cell proliferation

**FIGURE 8:**
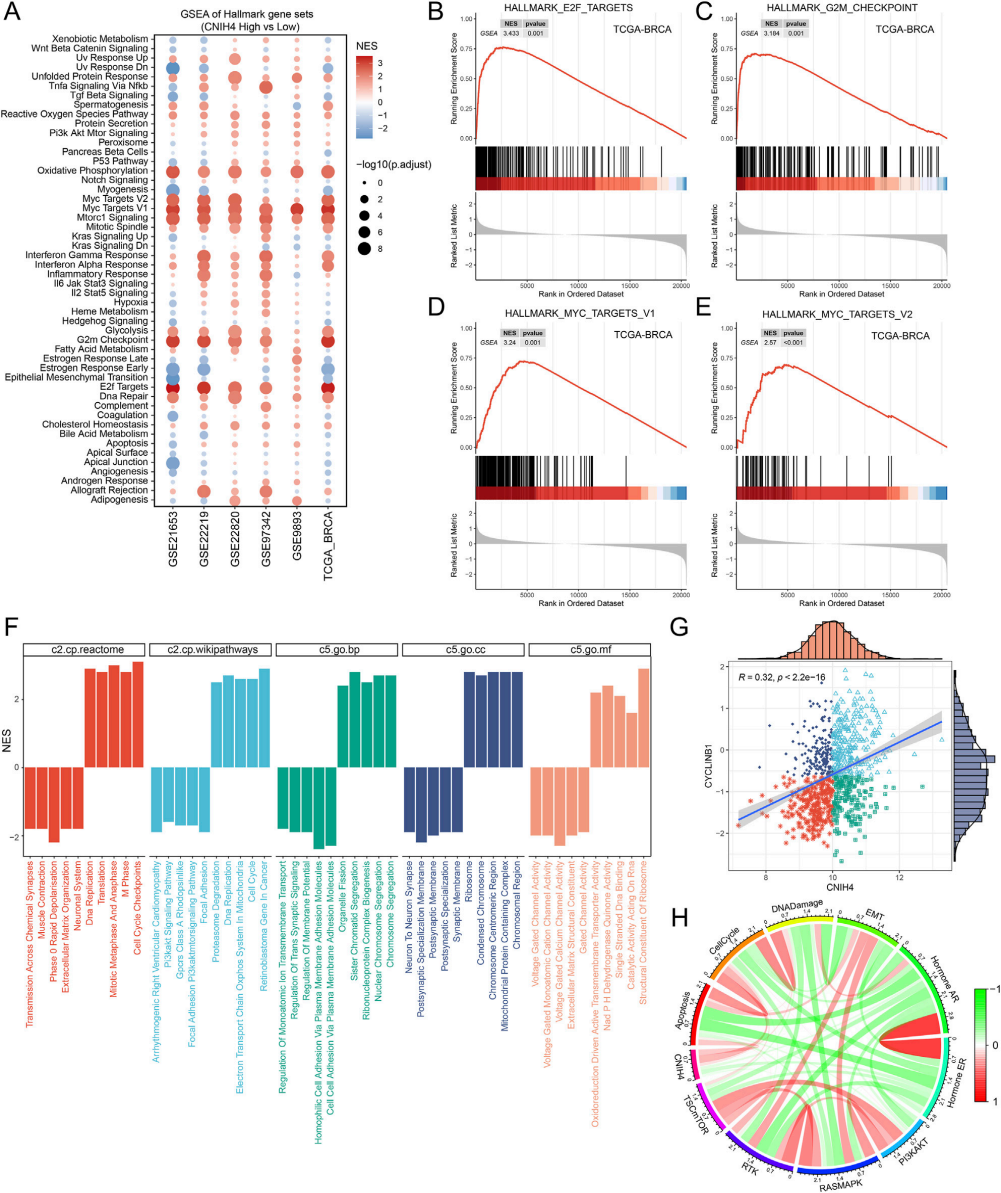
**(A)** Enrichment analysis of the Hallmark gene set and the KEGG metabolic gene set. NES values indicate significant enrichment of the target pathway in the CNIH4 low expression group, while positive NES values indicate enrichment in the high expression group. A p-value less than 0.05 and an adjusted p-value less than 0.25 are considered statistically significant; **(B–E)** CNIH4 is significantly enriched in multiple cell cycle-related pathways. The gene set is enriched at the upper end, indicating significant activation of the target pathway in the CNIH4 high expression group; **(F)** GSEA enrichment analysis of multiple gene sets validates the correlation between CNIH4 and cell cycle pathways. Different colors represent distinct gene sets. A downward direction in the bar graph indicates significant enrichment in the low-expression group, while an upward direction signifies enrichment in the high-expression group; **(G)** Correlation analysis between CNIH4 gene expression and TCPA-RPPA sequencing functional protein CYCLINB1. Each scatter point represents a sample, categorized into four groups based on median gene and protein values: high expression for both, high gene and low protein, low gene and high protein, and low expression for both; **(H)** Correlation of CNIH4 gene expression with pathway-level functional protein quantification by TCPA-RPPA sequencing. Darker red indicates a stronger positive correlation, while darker green signifies a stronger negative correlation. The width of the calculated line corresponds to the Spearman correlation coefficient.

The pan-cancer analysis revealed a robust association between CNIH4 and cell cycle regulation. Building on this finding, we conducted a more detailed investigation into the relationship between CNIH4 and the cell cycle in BRCA. Initially, gene set enrichment analysis (GSEA) of multiple BRCA datasets demonstrated a significant positive correlation between CNIH4 and cell cycle-related pathways (Figures 8A–E). Furthermore, GSEA, which included Gene Ontology (GO) biological processes (BP), molecular functions (MF), cellular components (CC), Reactome, and WikiPathways gene sets, underscored the substantial positive correlation between CNIH4 and cell cycle activity (Figure 8F). Additionally, differential expression analysis of the high and low expression groups of CNIH4 in BRCA identified a total of 267 significantly upregulated genes (Supplementary Figures 12A). Functional enrichment analysis further revealed that these genes were significantly enriched in several cell cycle pathways (Supplementary Figures 12B). Correlation analysis indicated that CNIH4 in BRCA was significantly and positively correlated with various cell cycle-related genes, including CCNB2, CDC20, and CDC25C (Supplementary Figures 12C–N). At the protein level, a significant positive correlation was observed between CNIH4 and the cell cycle-related protein CYCLINB1 in BRCA (Figure 8G). Furthermore, pathway analysis based on proteomic data confirmed a significant positive correlation between CNIH4 and cell cycle progression (Figure 8H).

**FIGURE 9:**
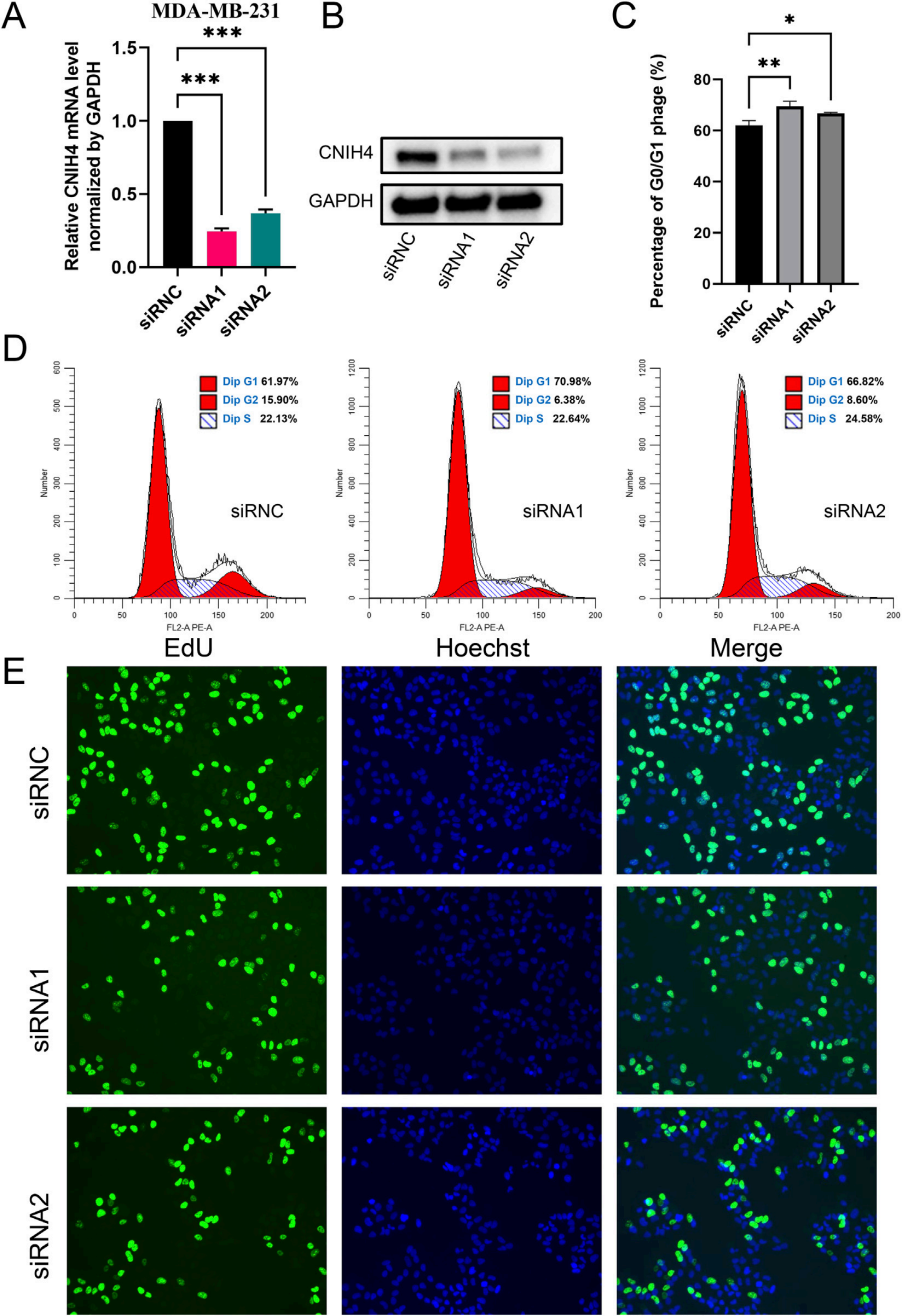
**(A)** PCR test for knockdown efficiency of CNIH4 gene in MDA-MB-231 cell line; **(B)** Western blotting to verify the knockdown of CNIH4 gene in MDA-MB-231 cell line; **(C)** Statistical analysis of the effects of CNIH4 knockdown on the cell cycle; **(D)** Flow cytometry analysis of the effect of CNIH4 knockdown on cell cycle; **(E)** EdU staining assay to evaluate the effect of CNIH4 knockdown on cell proliferation. *p < 0.05, **p < 0.01, ***p < 0.001. The results of EdU staining experiments are images generated at ×10 magnification, and the scale bar is 100 μm.

To experimentally validate the association between CNIH4 and the cell cycle in BRCA, we employed the BRCA cell line MDA-MB-231 for functional assays. Cells were transfected with two distinct siRNAs, followed by qRT-PCR and Western blotting to assess the effects on CNIH4 expression. Our findings indicated that both mRNA and protein levels of CNIH4 were reduced in the transfected groups relative to the control groups (Figures 9A, B). Cellular function experiments revealed that CNIH4 knockdown resulted in a significant accumulation of cells in the G0/G1 phase, suggesting inhibition of cell cycle progression and proliferation (Figures 9C, D). Moreover, the number of EdU-positive cells was markedly decreased after CNIH4 knockdown, indicating that CNIH4 deficiency could suppress cell proliferation (Figure 9E).

### 3.8 CNIH4 is significantly associated with drug sensitivity

**FIGURE 10:**
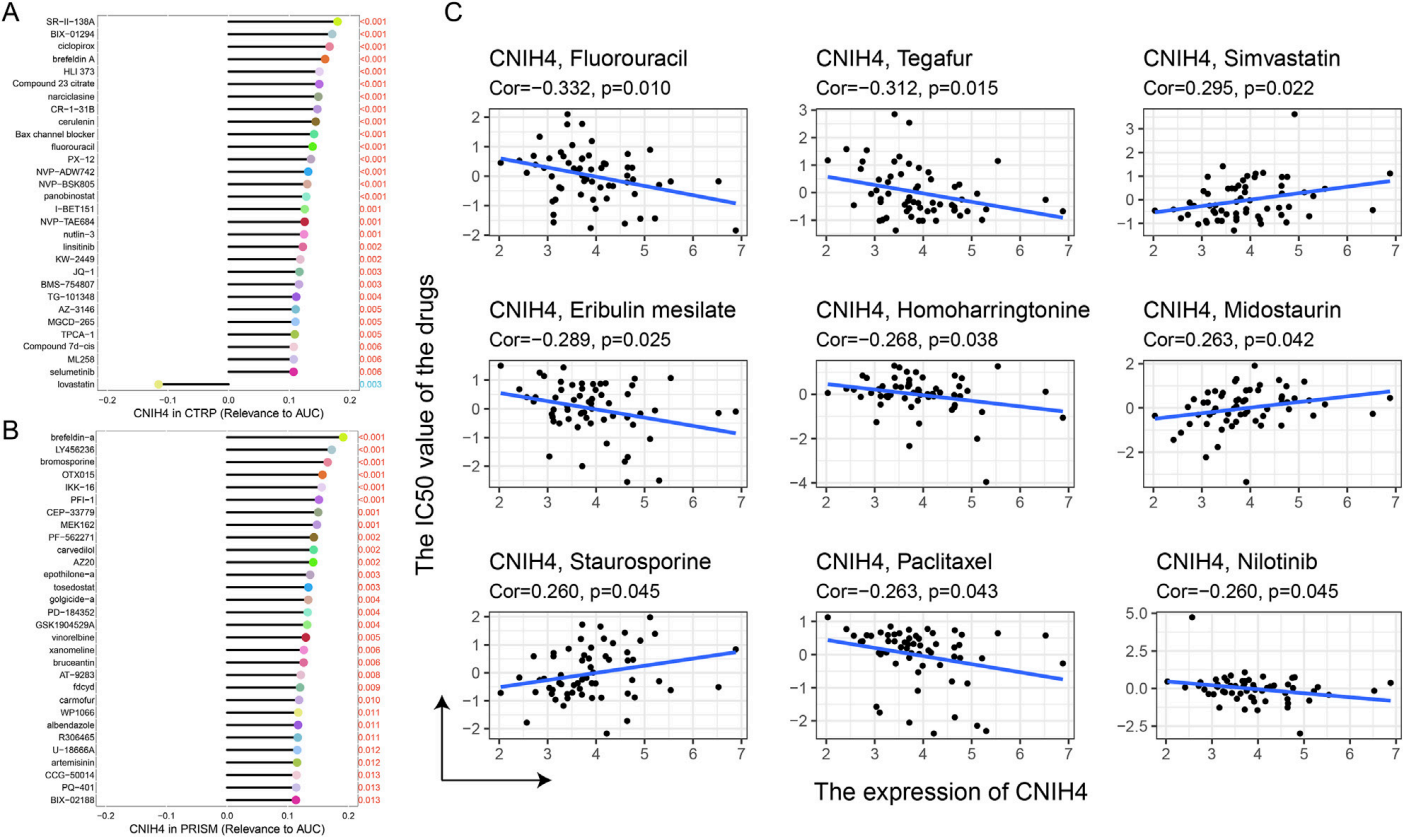
**(A, B)** Spearman correlation between CNIH4 gene and AUC values of drugs in CTRP and PRISM databases. The horizontal axis represents the Spearman correlation coefficient between the drug AUC value and gene expression, while the y-axis displays the top 30 drugs with the most significant p-values, each represented by a different color. A higher correlation coefficient corresponds to a longer stick in the lollipop chart; **(C)** Evaluation of the correlation between CNIH4 and drug sensitivity using the CellMiner database.

Analysis of drug sensitivity based on the CTRP and PRISM databases revealed a significant positive correlation between CNIH4 expression and the sensitivity to multiple kinase inhibitors, including selumetinib, ML258, and JQ-1. Additionally, there was a significant positive correlation with certain cell division inhibitors (e.g., epothilone-a), chemotherapeutic agents (e.g., fluorouracil), and some antifungal drugs (e.g., brefeldin A) (Figures 10A, B). Analysis using the CellMiner database indicated a significant positive correlation between CNIH4 and kinase inhibitors (e.g., Midostaurin and Staurosporine), and a significant negative correlation with drugs such as Eribulin mesilate, Tegafur, and Fluorouracil (Figure 10C).

## 4 Discussion

CNIH4 was initially identified as significantly overexpressed in Fibrolamellar carcinomas, suggesting its potential as an oncogene (Kannangai et al., 2007). Subsequent research revealed that CNIH4 interacts with newly synthesized GPCRs, regulating their export from the endoplasmic reticulum (Sauvageau et al., 2014). Moreover, CNIH4 has been shown to be significantly overexpressed in LIHC tissues and is significantly associated with gastric cancer cell proliferation, proposing it as a novel biomarker for ovarian cancer (Zhang et al., 2023; Wang et al., 2021; Kasavi, 2022). Additionally, CNIH4 is significantly associated with CESC and LGG (Xiao et al., 2023; Wang et al., 2023). While the tumor-promoting role of CNIH4 has been established in certain individual cancers, tumor heterogeneity necessitates a pan-cancer analysis to assess the prognostic variations and immune characteristics of CNIH4 across different cancer types.

This study elucidates the multifaceted role of CNIH4 in oncogenesis by conducting a comprehensive analysis of its expression patterns, genomic alterations, epigenetic modifications, prognostic implications, and interactions with the immune microenvironment across various cancers. Our findings indicate that CNIH4 is upregulated in multiple cancers, corroborating previous research that has established a link between elevated CNIH4 expression and tumor malignancy (Xiao et al., 2023). Through pan-cancer level expression pattern analysis, our study further substantiates CNIH4 as a universally associated oncogene. Genomic alterations are a common feature of tumors (Chang et al., 2023). Our analysis results showed a high mutation rate of CNIH4, especially in BRCA, suggesting that these variants may affect the expression and function of CNIH4, leading to tumorigenesis. CNV analysis showed that CNIH4 has common amplification mutations in various cancers, which may be related to its elevated expression in tumors. Notably, an increase in CNIH4 copy number was positively correlated with its mRNA expression level, which may drive further overexpression of CNIH4 in tumors and consequently impact tumor biology. DNA methylation plays an important role in the occurrence and development of tumors, and its abnormal changes are closely related to the early detection, diagnosis, treatment, and prognosis of cancer (Papanicolau-Sengos and Aldape, 2022). Our results reveal dysregulation of CNIH4 methylation levels in multiple tumors, which may affect its transcriptional activity and protein expression, emphasizing the importance of genomic and epigenetic regulation in CNIH4 expression. Additionally, high CNIH4 expression was associated with poor prognosis in various cancers, reinforcing the critical role of CNIH4 in oncogenesis and suggesting its potential as an independent prognostic marker. Functional enrichment analysis demonstrated significant associations between CNIH4 and multiple cancer-related pathways, notably those involved in cell cycle regulation and DNA repair, which are pivotal in tumor cell proliferation and survival. High CNIH4 expression may enhance the activity of these pathways, thereby promoting tumor development. Previous studies have emphasized the interaction between CNIH4 and TGFα (Mishra et al., 2019). TGFα, a well-established driver of EMT and cancer stem cell properties, plays a crucial role in cancer metastasis and therapeutic resistance. CNIH4 may amplify the oncogenic effects of TGFα signaling, thereby advancing tumor progression and metastasis. This finding underscores the potential role of CNIH4 in regulating the tumor microenvironment and influencing cancer development at the molecular level. Our study also unveiled the immune signature of CNIH4 in pan-cancer, highlighting its correlation with immune subtypes, immune-related genes, and immune cell infiltration. These findings resonate with the complex interplay between immune suppression and activation observed in the tumor microenvironment (Marzagalli et al., 2019). The association of high CNIH4 expression with IFN-γ-dominated immune subtypes suggests a role for CNIH4 in modulating immune responses within the tumor microenvironment. Furthermore, the positive correlation of CNIH4 with multiple immune checkpoints may influence tumor responsiveness to immunotherapy, offering novel insights into the role of CNIH4 in the tumor immune microenvironment and its potential as a target for immunotherapeutic strategies.

Our study specifically investigated the association between CNIH4 and BRCA. Our findings revealed that CNIH4 expression in BRCA was significantly higher than in normal cells, particularly in the MDA-MB-231 cell line, suggesting a pivotal role for CNIH4 in the malignant transformation and tumor progression of BRCA. Clinical feature analysis indicated that CNIH4 expression in BRCA correlated with tumor malignancy and stage. Our study contributes novel evidence for the significant role of CNIH4 in BRCA development by examining CNIH4 expression patterns at the single-cell and spatial transcriptome levels. GSEA analysis results highlighted a robust association between CNIH4 and the cell cycle. In BRCA, CNIH4 was significantly and positively correlated with multiple cell cycle-related genes and proteins, suggesting a regulatory role for CNIH4 in cell cycle control and tumor cell proliferation. Functional experimental outcomes demonstrated that CNIH4 knockdown led to an accumulation of cells in the G0/G1 phase, indicating that CNIH4 may promote breast cancer cell proliferation by modulating the cell cycle. Given the elevated CNIH4 expression in breast cancer and its impact on the cell cycle and proliferation, CNIH4 could emerge as a potential therapeutic target. Drug sensitivity analysis revealed a significant correlation between CNIH4 expression and the sensitivity to various drugs. Entinostat, a histone deacetylase inhibitor used to treat various breast cancers, including HER2-positive and triple-negative breast cancer (Sidiropoulos et al., 2022; Trapani et al., 2017), was identified as a potential drug targeting CNIH4 using the Cmap database. However, further studies are needed to verify its effectiveness. Future research could focus on developing CNIH4-targeted therapeutic strategies to inhibit breast cancer cell proliferation and tumor progression.

This study presents a comprehensive analysis of CNIH4, examining its expression patterns, genomic alterations, epigenetic modifications, prognostic significance, and interactions with the immune microenvironment across a spectrum of cancers. Our results underscore the multifaceted role of CNIH4 in oncogenesis and posit that CNIH4 could emerge as a promising biomarker and therapeutic target. Further research is warranted to elucidate the molecular mechanisms underlying CNIH4’s role in tumorigenesis and to translate these insights into effective clinical interventions.

## Data Availability

The original contributions presented in the study are included in the article/Supplementary Material, further inquiries can be directed to the corresponding author.
