# Supplementary material for: Deciphering the role of CNIH4 in pan-cancer landscapes and its significance in breast cancer progression
Source: Front Genet. 2025 Feb 20;16:1536620. doi: 10.3389/fgene.2025.1536620 (PMC11882561; doi:10.3389/fgene.2025.1536620)
Supplement: Supplementary file 1 [file Table1.docx]

Supplementary Material

Deciphering the Role of CNIH4 in Pan-Cancer Landscapes and Its Significance in Breast Cancer Progression

Yao Xu^1#^, Zengzhen Lai^1#^, Chaolin Li^1*^

^1^Jinniu Maternity and Child Health Hospital of Chengdu, Sichuan Province, China.

^#^These authors contributed equally and should be considered co-first authors.

^*^Corresponding Author: Chaolin Li (kone_lcl@foxmail.com).

# Supplementary Figures


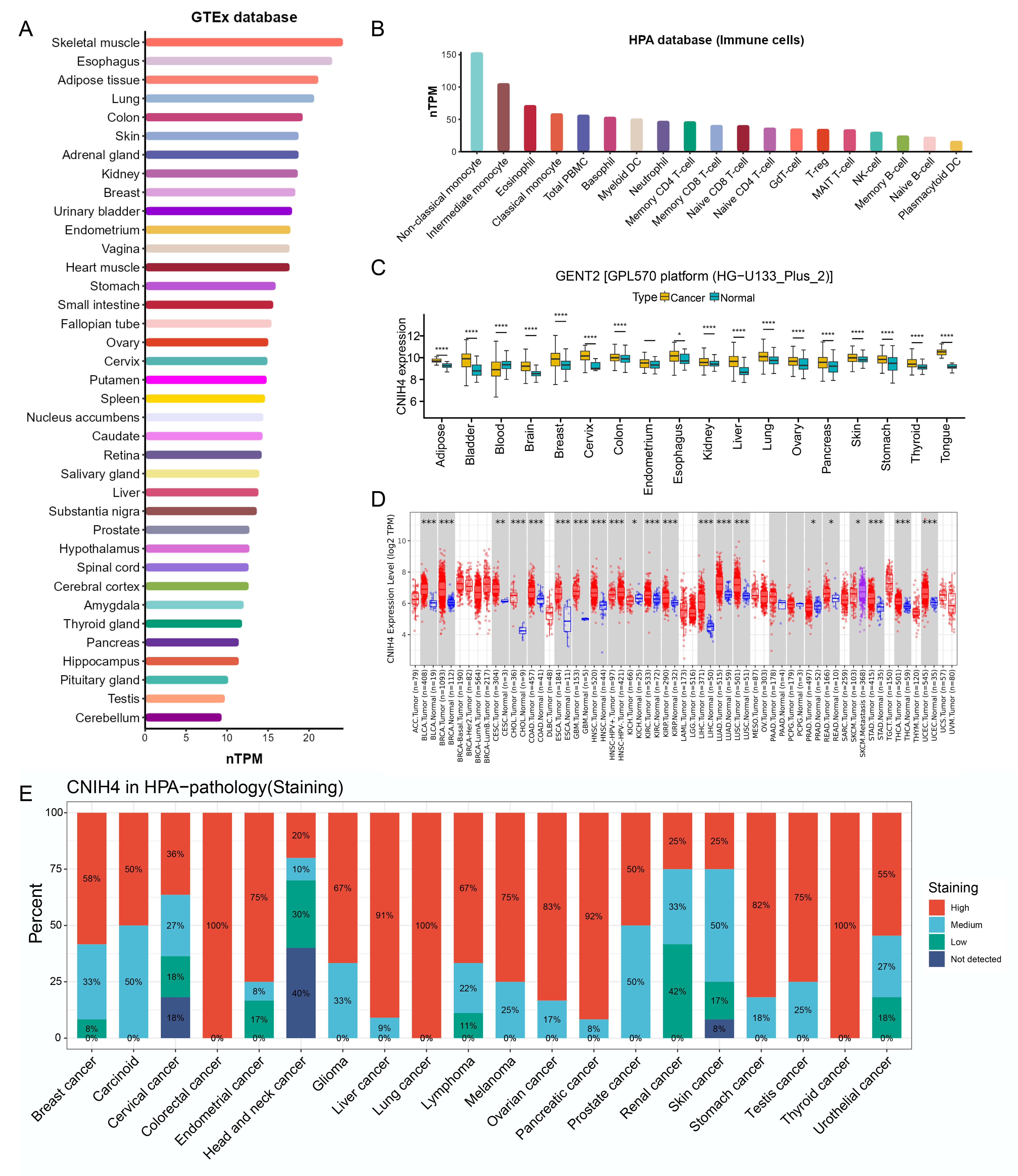


**Supplementary Figure 1**. (A) Evaluation of CNIH4 expression levels in normal human tissues based on the GTEx database; (B) Evaluation of CNIH4 expression level in immune cells based on HPA database; (C) Verification of differential expression of CNIH4 based on GENT2 database; (D) Verification of differential expression of CNIH4 based on TIMER2.0 database; (E) HPA immunohistochemical staining results.

**
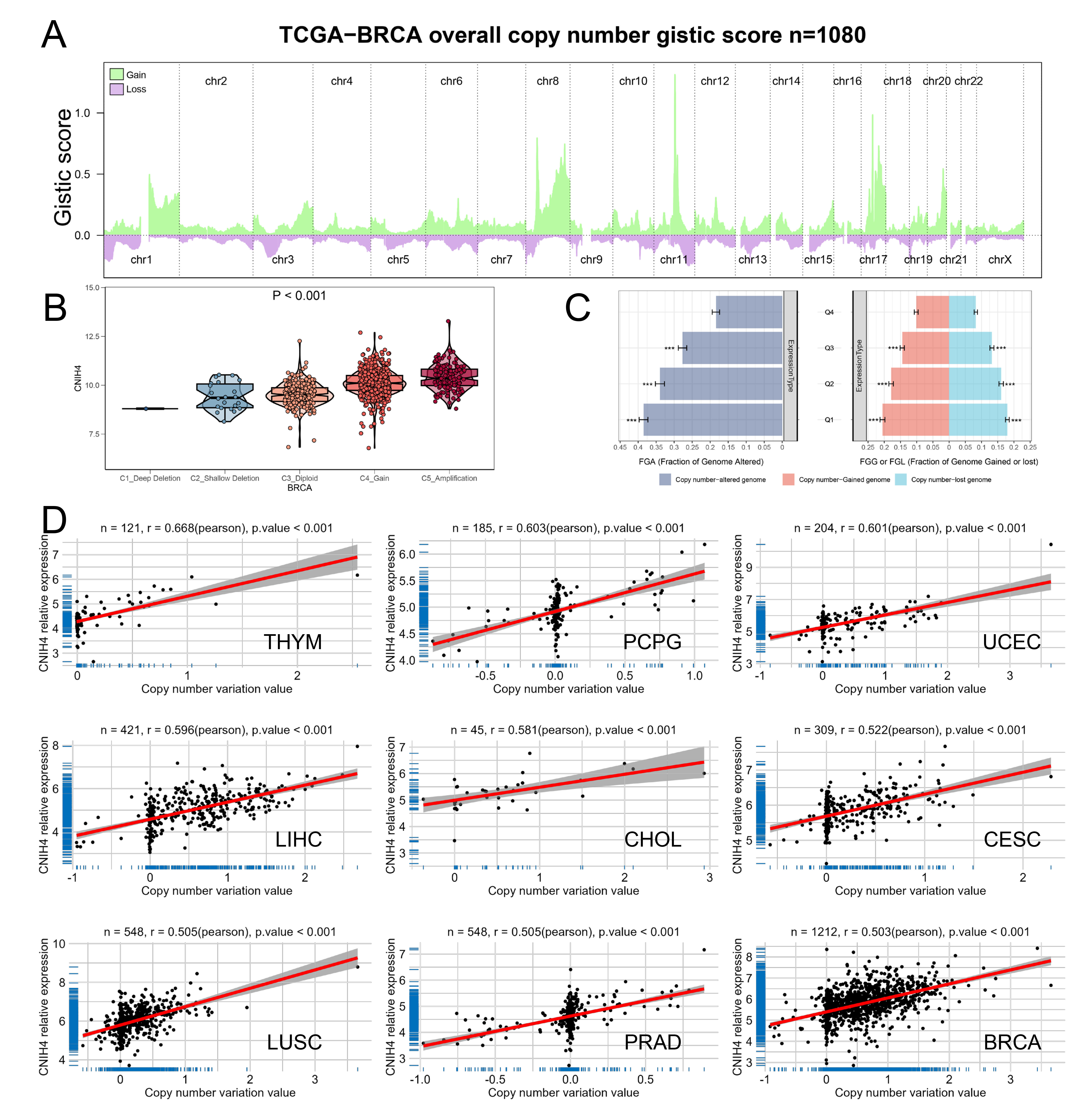
**

**Supplementary Figure 2**. (A) TCGA-BRCA copy number gistic2 scoring; (B) Differences in CNIH4 gene expression in different BRCA copy number variation types; (C) V Percentage of FGL, FGG, and FGA in CNIH4 expression subsets for BRCA; (D) Scatter plot of correlation between CNIH4 copy number variation and mRNA expression in pan-cancer.

**
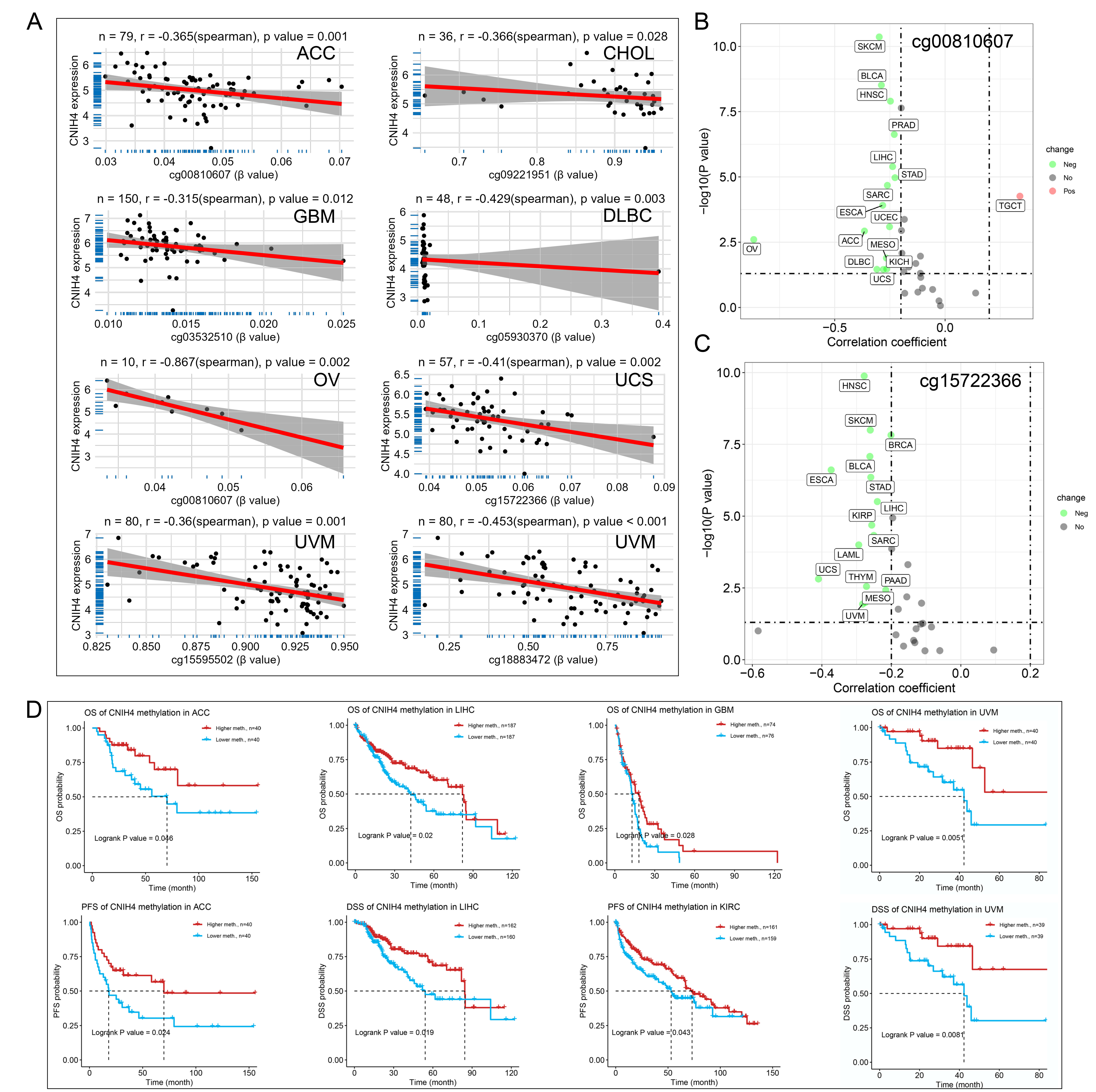
**

**Supplementary Figure 3**. (A) Scatter plot of correlation between CNIH4 methylation level and mRNA expression in pan-cancer; (B, C) Scatter plot of correlation between methylation level and mRNA expression of cg00810607 and cg15722366 probes in Pan-Cancer; (D) KM survival analysis revealed the correlation between CNIH4 methylation level and clinical prognosis.

**
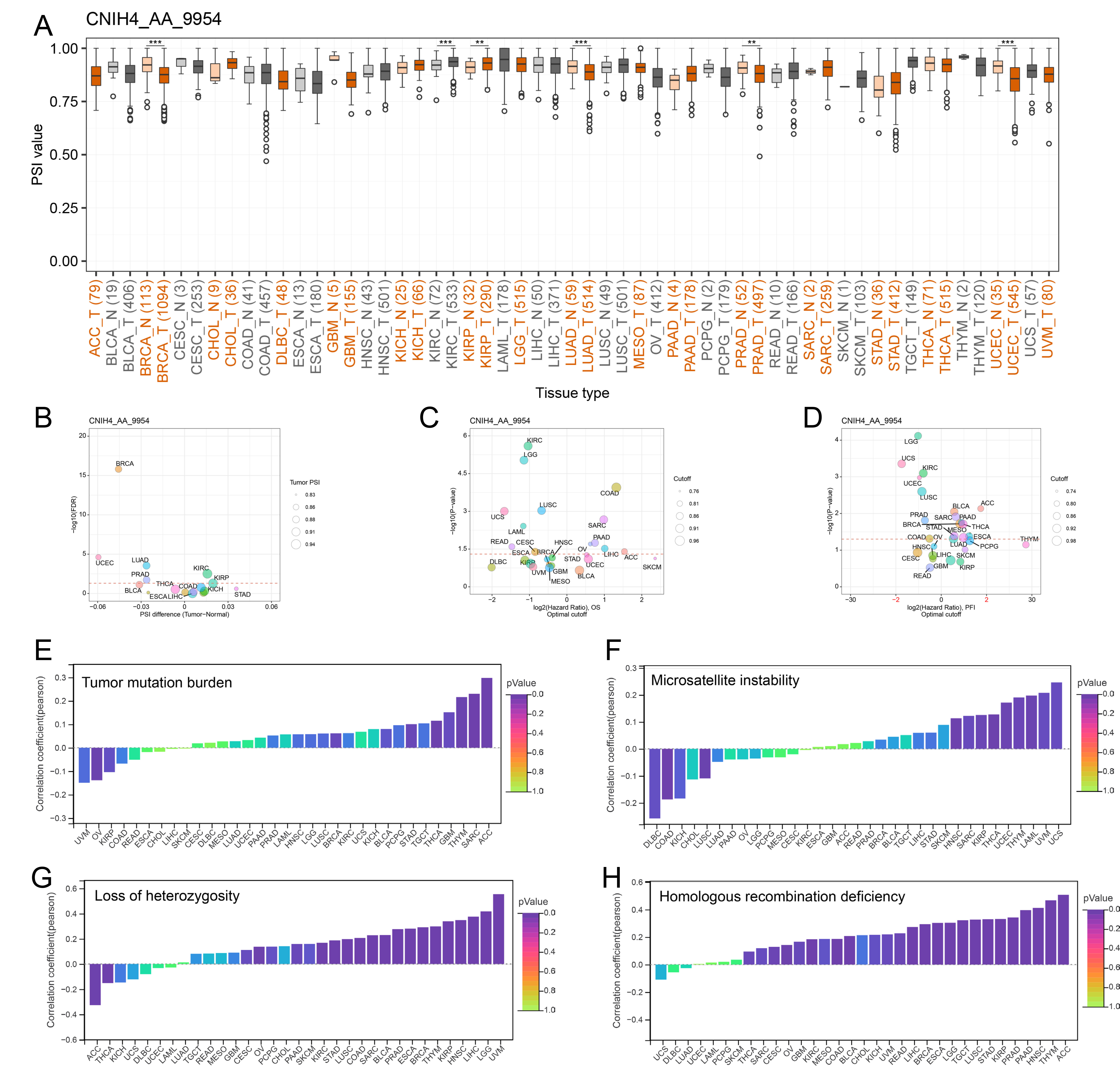
**

**Supplementary Figure 4**. (A) Alternative splicing events of CNIH4_AA_9954 in pan-cancer; (B-D) PSI differences when comparing tumors and corresponding healthy or adjacent tissues and the association between CNIH4_ AA_9954 events and prognosis; (E-H) Correlation analysis between CNIH4 expression and TMB, MSI, LOH and HRD in pan-cancer.

**
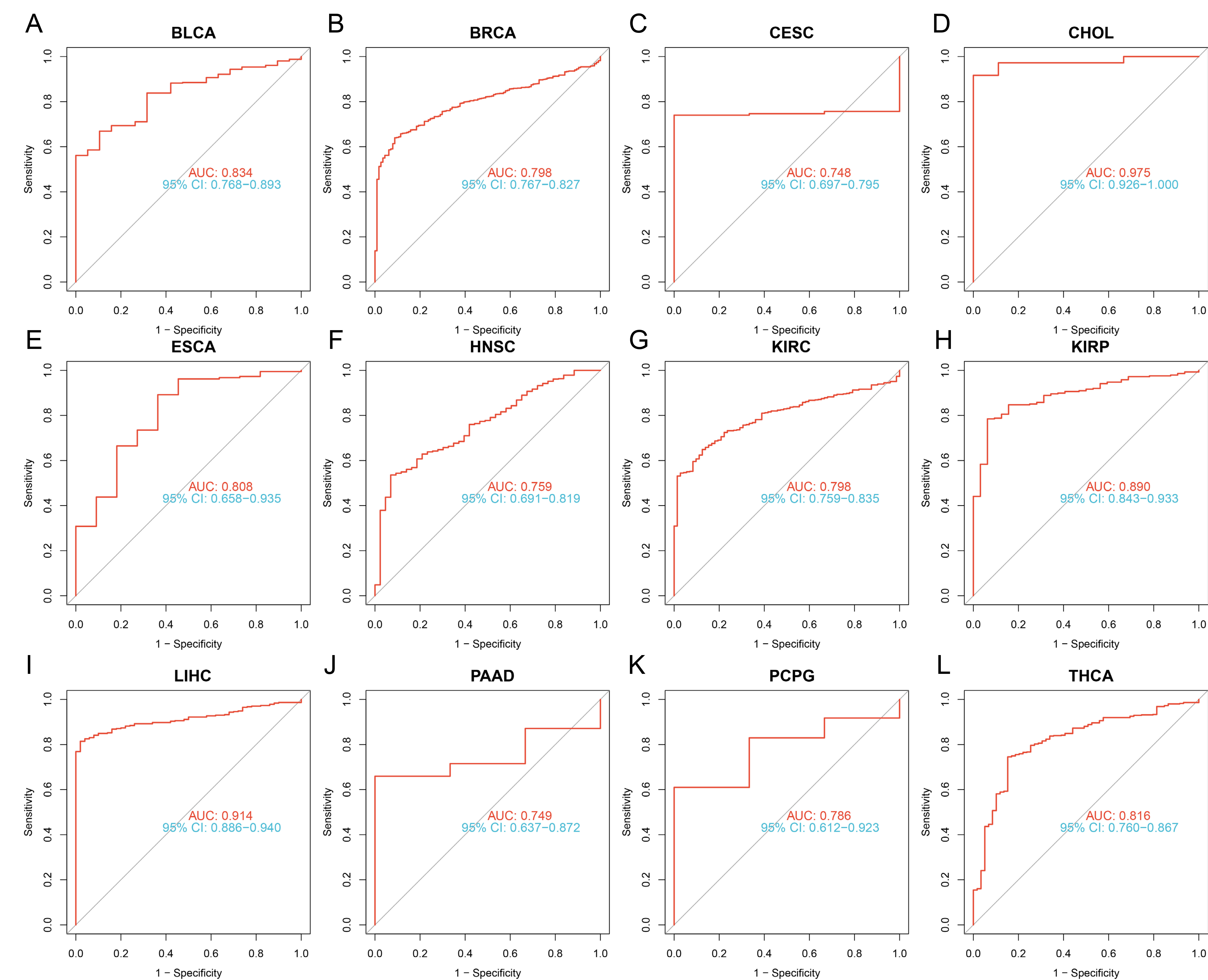
**

**Supplementary Figure 5**. (A-L) ROC analysis to evaluate the potential of CNIH4 as a diagnostic marker.


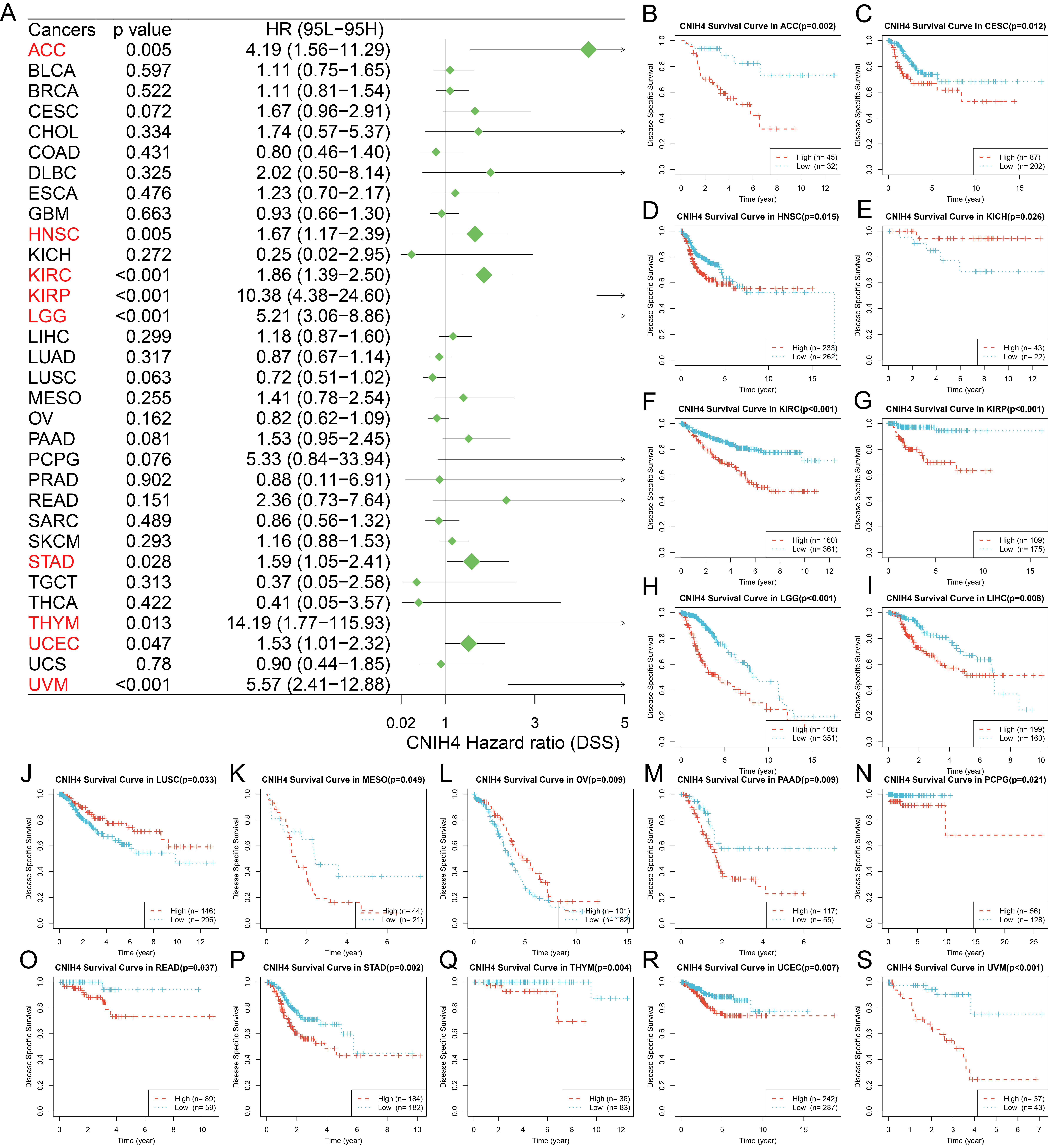


**Supplementary Figure 6**. (A) Univariate Cox survival analysis of DSS in pan-cancer; (B-S) Kaplan-Meier survival analysis of DSS in Pan-Cancer.


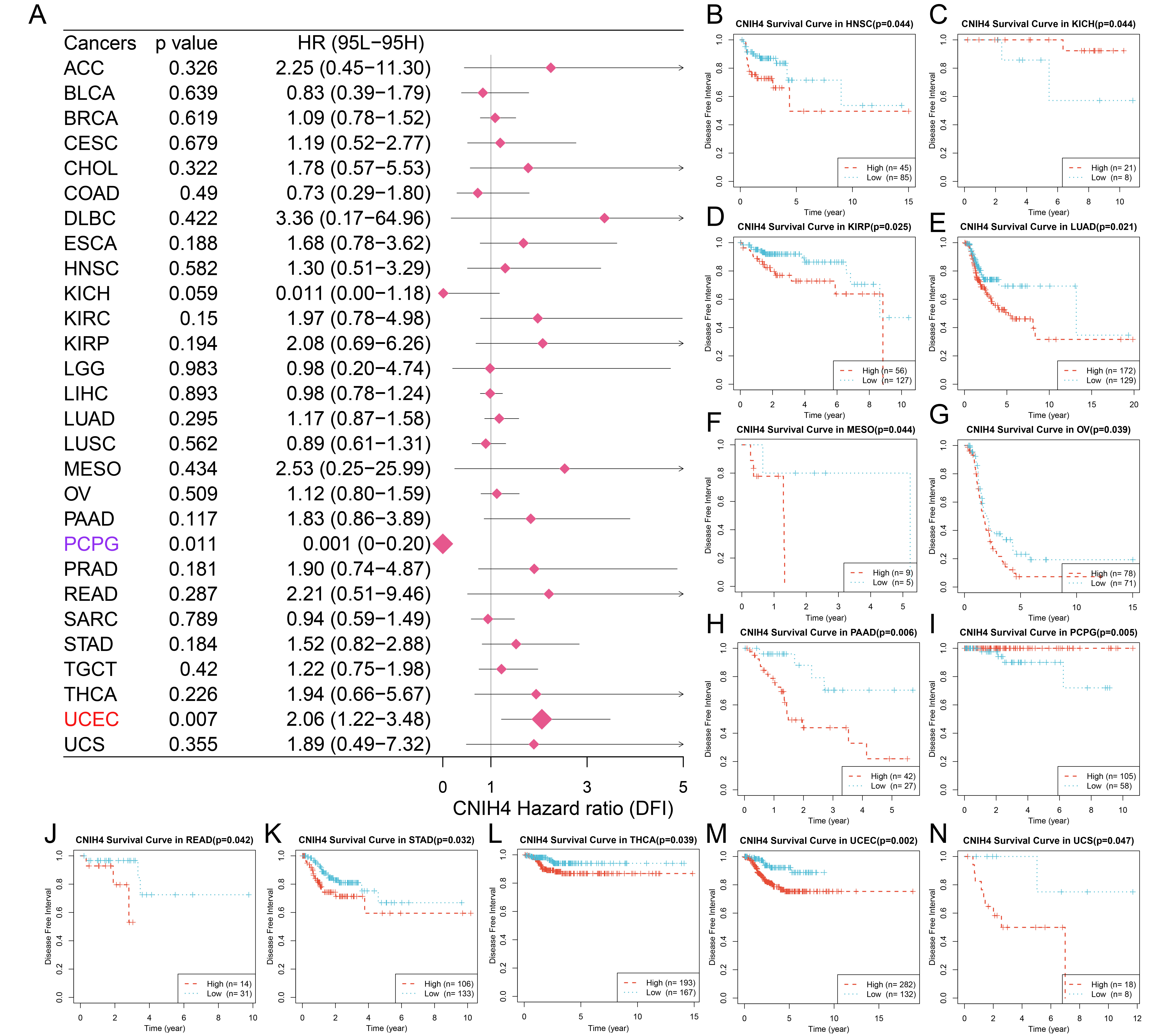


**Supplementary Figure 7**. (A) Univariate Cox survival analysis of DFI in pan-cancer; (B-N) Kaplan-Meier survival analysis of DFI in Pan-Cancer.


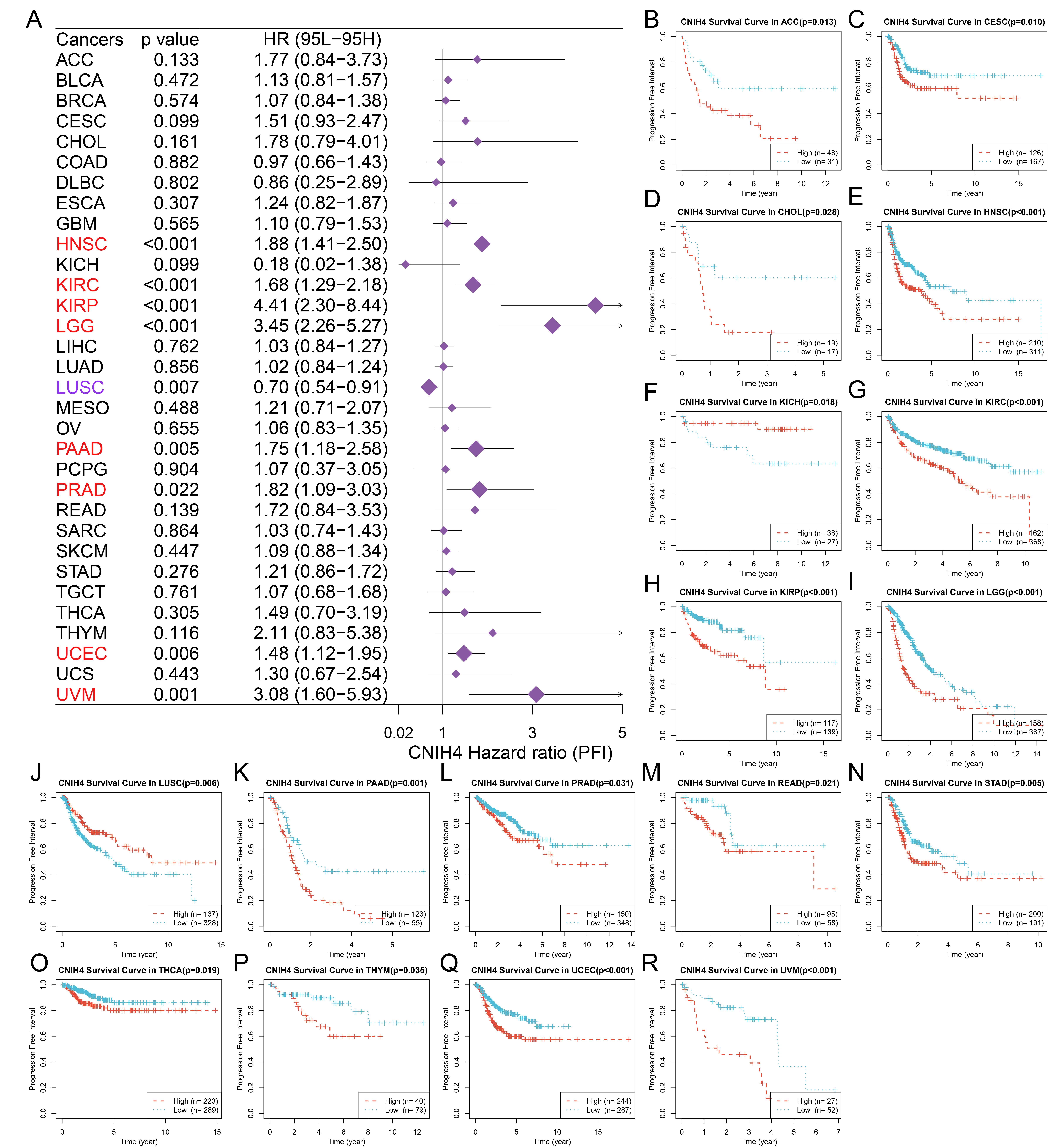


**Supplementary Figure 8**. (A) Univariate Cox survival analysis of PFI in pan-cancer; (B-R) Kaplan-Meier survival analysis of PFI in Pan-Cancer.


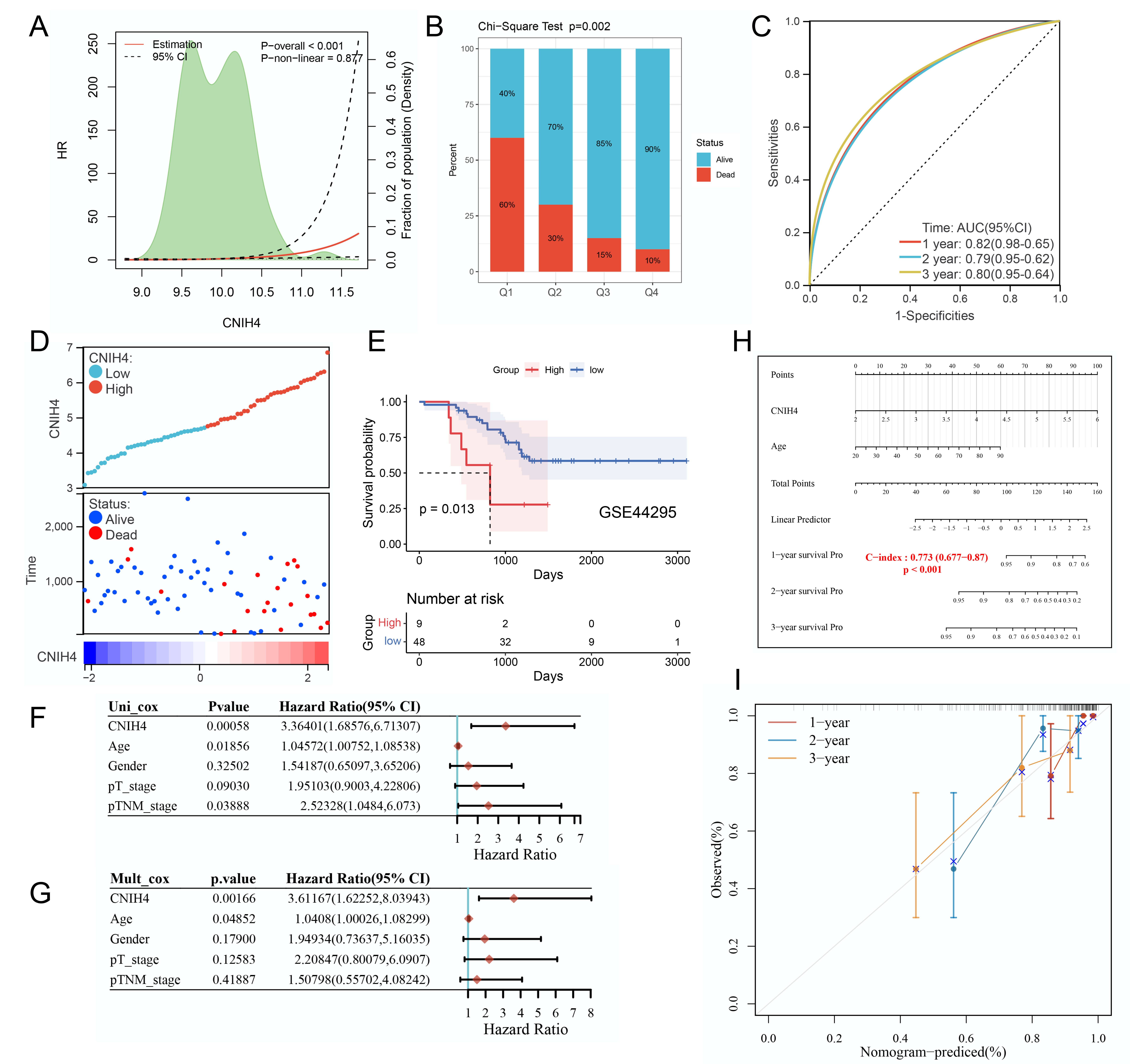


**Supplementary Figure 9**. (A) Restricted cubic spline method was used to explore whether the risk of CNIH4 gene on OS in UVM is nonlinear; (B) Chi-square test on the number of UVM patients alive and dead in different CNIH4 groups; (C) ROC analysis evaluated the predictive performance of CNIH4 for 1-year, 2-year, and 3-year survival rates of UVM patients; (D) Linkage diagram between CNIH4 expression and survival status of UVM patients; (E) GSE44295 dataset validates the prognostic value of CNIH4 in UVM; (F, G) Univariate and multivariate analyses evaluated the potential of CNIH4 as an independent prognostic factor for UVM; (H) A nomogram was constructed based on CNIH4 gene and clinical characteristics to predict the 1-, 2-, and 3-year survival rates of UVM patients; (I) Calibration curves were used to evaluate the predictive performance of the model.


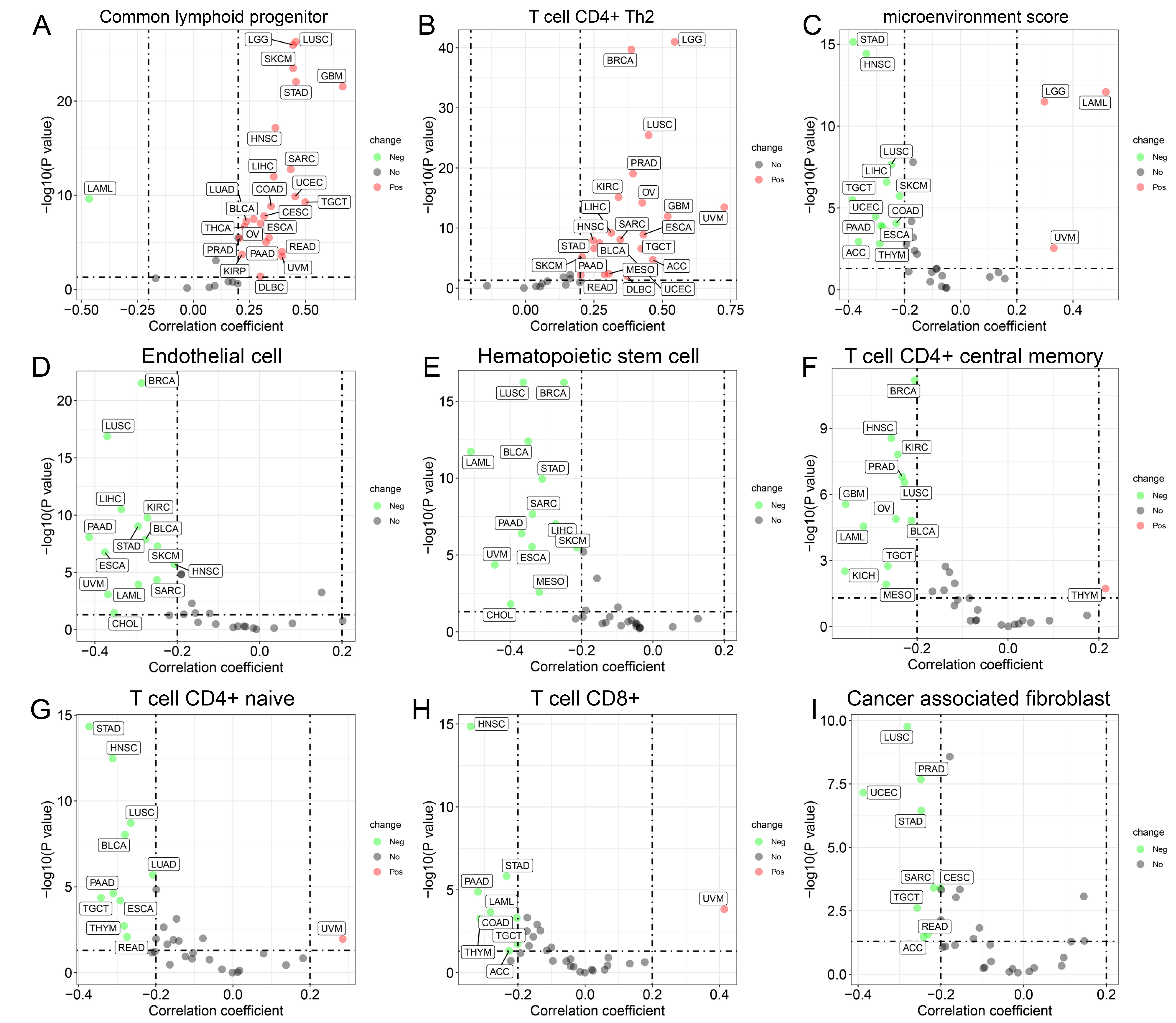


**Supplementary Figure 10**. (A-I) Evaluation of the correlation between different immune cell infiltration states and CNIH4 in pan-cancer.


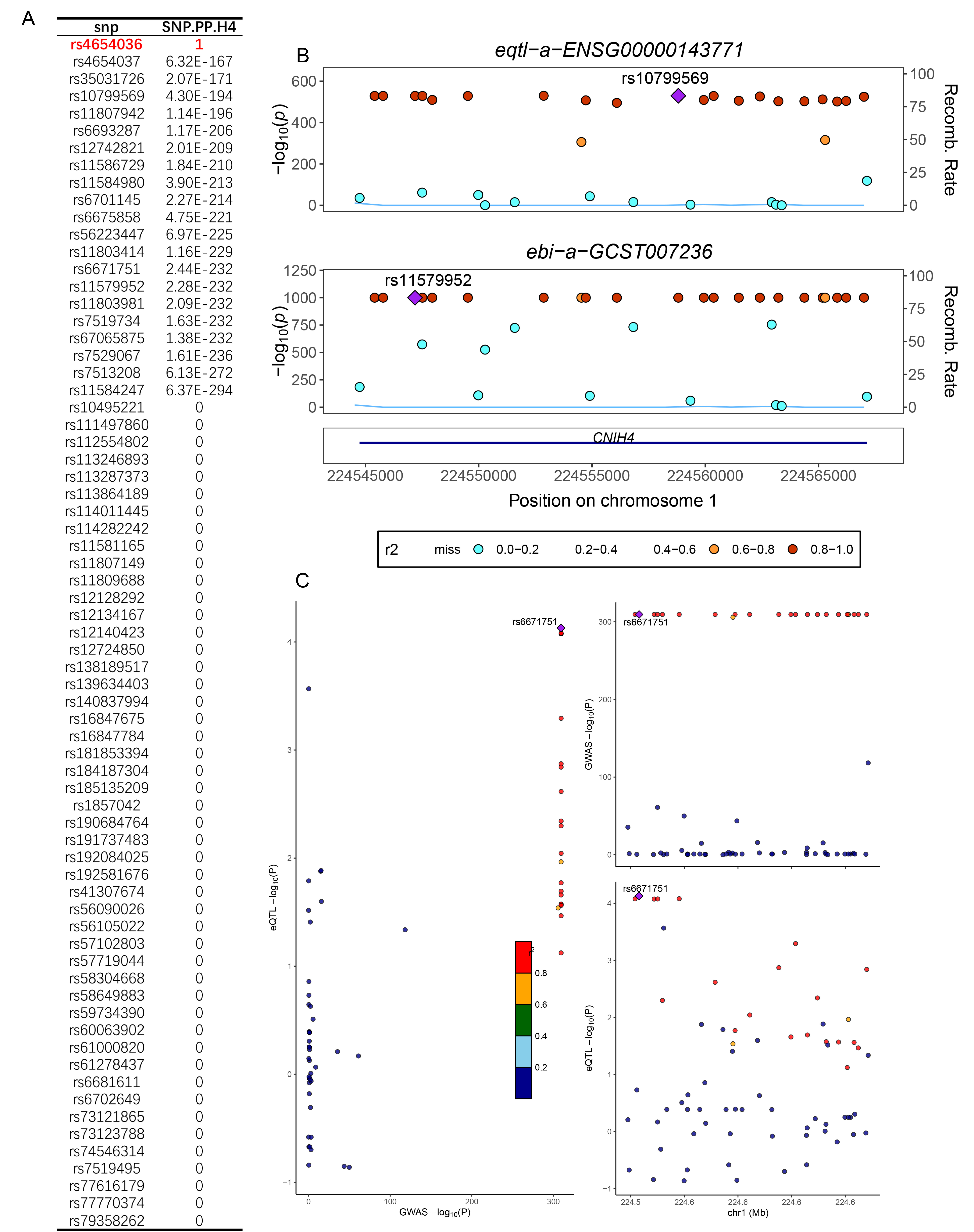


**Supplementary Figure 11**. (A) Evaluate whether different SNP sites of CNIH4 and BRCA in the ebi-a-GCST007236 dataset share genetic causal variants; (B) gassocplo package visualizes eQTL-GWAS co-localization results; (C) locuscompare package visualizes eQTL-GWAS co-localization results.


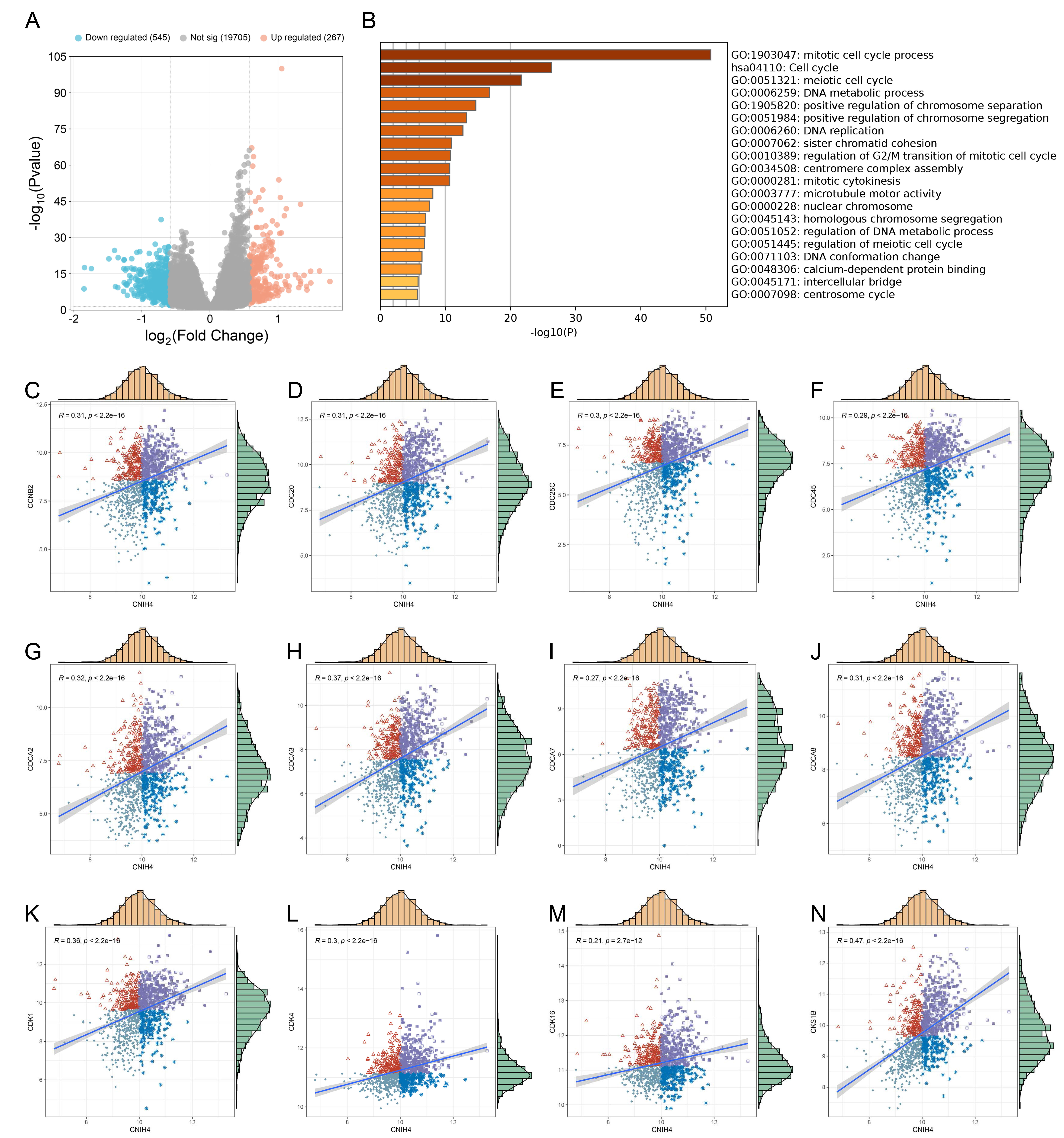


**Supplementary Figure 12**. (A) Volcano plot showing differentially expressed genes between high and low expression groups of CNIH4 in BRCA; (B) Functional enrichment analysis showed that the 267 significantly upregulated genes were mainly enriched in multiple cell cycle pathways; (C-L) Correlation analysis between CNIH4 and cell cycle-related genes in BRCA.
